# Exploring Chickpea Germplasm Diversity for Broadening the Genetic Base Utilizing Genomic Resourses

**DOI:** 10.3389/fgene.2022.905771

**Published:** 2022-08-04

**Authors:** Rajesh Kumar Singh, Charul Singh, Ambika, B. S. Chandana, Rohit K. Mahto, Ranjana Patial, Astha Gupta, Vijay Gahlaut, Gayacharan, Aladdin Hamwieh, H. D. Upadhyaya, Rajendra Kumar

**Affiliations:** ^1^ Indian Agricultural Research Institute (ICAR), New Delhi, India; ^2^ University School of Biotechnology, Guru Gobind Singh Indraprastha University, New Delhi, India; ^3^ Department of Genetics and Plant Breeding, University of Agricultural Sciences, Bangalore, Bangalore, India; ^4^ Department of Agricultural Sciences, Chandigarh University, Mohali, India; ^5^ School of Agricultural Sciences, Sharda University, Greater Noida, India; ^6^ Institute of Himalayan Bioresource Technology (CSIR), Pālampur, India; ^7^ National Bureau of Plant Genetic Resources (ICAR), New Delhi, India; ^8^ International Center for Agriculture Research in the Dry Areas (ICARDA), Giza, Egypt; ^9^ Department of Entomology, International Crops Research Institute for the Semi-Arid Tropics (ICRISAT), Hyderabad, India; ^10^ Plant Genome Mapping Laboratory, University of Georgia, Athens, GA, United States

**Keywords:** broadening the genetic base, cicer, genetic diversity (GD), gene editing, multiple resistance, omics, QTL mapping, wild chickpea utilization

## Abstract

Legume crops provide significant nutrition to humans as a source of protein, omega-3 fatty acids as well as specific macro and micronutrients. Additionally, legumes improve the cropping environment by replenishing the soil nitrogen content. Chickpeas are the second most significant staple legume food crop worldwide behind dry bean which contains 17%–24% protein, 41%–51% carbohydrate, and other important essential minerals, vitamins, dietary fiber, folate, β-carotene, anti-oxidants, micronutrients (phosphorus, calcium, magnesium, iron, and zinc) as well as linoleic and oleic unsaturated fatty acids. Despite these advantages, legumes are far behind cereals in terms of genetic improvement mainly due to far less effort, the bottlenecks of the narrow genetic base, and several biotic and abiotic factors in the scenario of changing climatic conditions. Measures are now called for beyond conventional breeding practices to strategically broadening of narrow genetic base utilizing chickpea wild relatives and improvement of cultivars through advanced breeding approaches with a focus on high yield productivity, biotic and abiotic stresses including climate resilience, and enhanced nutritional values. Desirable donors having such multiple traits have been identified using core and mini core collections from the cultivated gene pool and wild relatives of Chickpea. Several methods have been developed to address cross-species fertilization obstacles and to aid in inter-specific hybridization and introgression of the target gene sequences from wild *Cicer* species. Additionally, recent advances in “Omics” sciences along with high-throughput and precise phenotyping tools have made it easier to identify genes that regulate traits of interest. Next-generation sequencing technologies, whole-genome sequencing, transcriptomics, and differential genes expression profiling along with a plethora of novel techniques like single nucleotide polymorphism exploiting high-density genotyping by sequencing assays, simple sequence repeat markers, diversity array technology platform, and whole-genome re-sequencing technique led to the identification and development of QTLs and high-density trait mapping of the global chickpea germplasm. These altogether have helped in broadening the narrow genetic base of chickpeas.

## 1 Introduction

Grain legumes are a key component of the agricultural ecosystem. These plants are a chief member of the most diverse and ecologically crucial botanical families. Legumes play a vital role in crop rotations or intercropping schemes as these plants are capable of nitrogen assimilation through symbiotic relationship with rhizobia. Chickpea (*Cicer arietinum*) is the second most important grain legume after dry bean (*Phaseolus vulgaris* L.). Chickpeas have eight pairs of homologous chromosomes (2n = 16) with an estimated genome size of 738 Mb and 28,269 annotated genes (Varshney et al., 2013). The cultivated chickpea is believed to be originated in the Anatolia of Turkey (Van der Maesen, 1984). Vavilov denominated two primary centers of origin for chickpea viz., southwest Asia (Afghanistan) and the Mediterranean with the secondary center of origin as Ethiopia. Since ancient’s times, legumes have been grown for human subsistence. Globally, India is the largest producer and consumer of pulse crops. Pulses are the major source of carbohydrates, proteins, lipids, vitamins, and minerals for people across the globe (Aykroyd and Doughty, 1982). Pulses complement the nutritional quality, bioavailability of nutrients, when consumed along with cereals. Pulses provide 22–24% of protein, which is about twice the amount of wheat and three times the rice. Pulses are one of the cheapest sources of protein and play a very significant role in sustaining nutritional requirements in developing and economically poor countries. They have a low glycemic index (GI) and elicit only a moderate postprandial glycemic response after consumption. As a result, incorporating legumes into one’s diet is advised for glycemic-influenced diabetes control (Rizkalla et al., 2002).

Chickpea is the major source of food and nutrition in the semi-arid tropics. In comparison to other pulses, chickpeas are a rich source of protein and carbohydrates, accounting 80% to the whole mass of dried seeds (Geervani, 1991; Chibbar et al., 2010). Chickpea is high in dietary fiber (DF), vitamins, and minerals and is known to lower low-density lipoprotein (Wood and Grusak, 2007). Chickpea has the highest quantity of total DF amongst pulses, which ranges from 18 to 22 g/100 g of raw seed (Aguilera et al., 2009). The soluble and insoluble DF contents of chickpea raw seeds are about 4–8 and 10–18 g/100 g, respectively (Dalgetty and Baik, 2003). It has been demonstrated that chickpeas have more bioavailable protein than other legumes (Sánchez-Vioque et al., 1999; Yust et al., 2003). The changes in protein content of pre- and post-dehulled chickpea dried seeds are observed which range from 17%–22% and 25.3%–28.9%, respectively (Hulse, 1991; Badshah et al., 2003). Raw chickpea seeds have a total fat content ranging from 2.70 to 6.48% (Kaur et al., 2005; Alajaji and El-Adawy, 2006). On an average, raw chickpea seeds give 5.0 mg/100 g Fe, 4.1 mg/100 g Zn, 138 mg/100 g Mg, and 160 mg/100 g Ca. Chickpea is an inexpensive, rich source of folate and tocopherol (Ciftci et al., 2010). The major carotenoids, viz*.*, β-carotene, lutein, zeaxanthin, β-cryptoxanthin, lycopene, and α-carotene are also found in chickpea.

Globally two types of chickpea cultivars desi or microsperma and Kabuli or macrosperma are cultivated. Generally, Kabuli chickpea is predominantly cultivated in temperate regions like the Mediterranean region that includes Western Asia, Southern Europe, and Northern Africa. However, desi chickpea is raised mainly in the semi-arid tropics (Malhotra et al., 1987; Muehlbauer and Singh, 1987) such as Ethiopia and the Indian sub-continent. In general, desi types are characterized by small seeds, angular shape with a rough surface having a dark seed coat and flowers of pink or purple color due to the presence of anthocyanin pigment, whereas Kabuli types are bold seeded owl shaped with smooth surface have beige seed coat and bear white color flowers because of lack of anthocyanin pigment (Pundir et al., 1985). Desi-type chickpeas are generally early maturing and high yielding than the Kabuli type. The desi chickpea is the predominant form cultivated in India occupying approximately 80–85% and the Kabuli chickpea occupies the remaining 15–20% of the total area and production. The chickpea draft genome sequences are already reported for desi (Jain et al., 2013) and Kabuli (Varshney et al., 2013) types.

**FIGURE 1:**
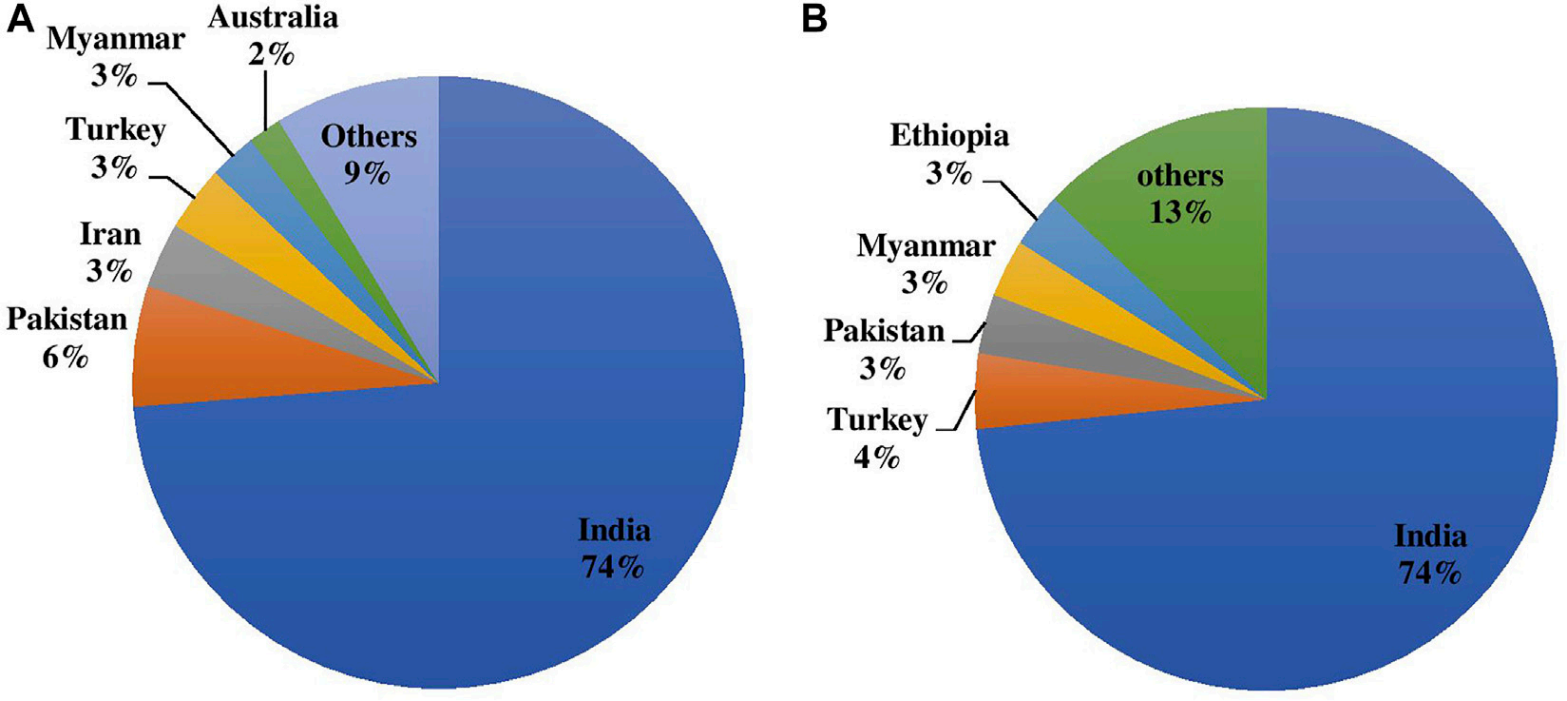
**(A)** Area and **(B)** Production of chickpea during 2020 in major producing countries in the world.

Chickpeas are majorly grown as rainfed crops since they require less irrigation than other competitive crops such as cereals. However, it can be grown in a wide range of soils and agro-climatic conditions. Chickpea contributes to farming systems’ long-term survival as it plays important roles in crop rotation, mixed and intercropping, soil fertility maintenance through nitrogen fixation, and the release of soil-bound phosphorus; overall it improves the soil ecosystem. Globally, chickpea is grown on 14.842 m ha with an annual production volume of 15.083 m tones having a productivity average of 1,016 kg/ha. Indian contribution to the globe is 73.769% (10.949 m ha) in terms of area and 73.456% (11.080 m tones) production as depicted in Figures 1A,B with average productivity of 1,012 kg/ha (FAOSTAT 2020). Pakistan, Turkey, Australia, Myanmar, Ethiopia, Iran, Mexico, Canada, China, and the United States are among the other significant chickpea producers.

**FIGURE 2:**
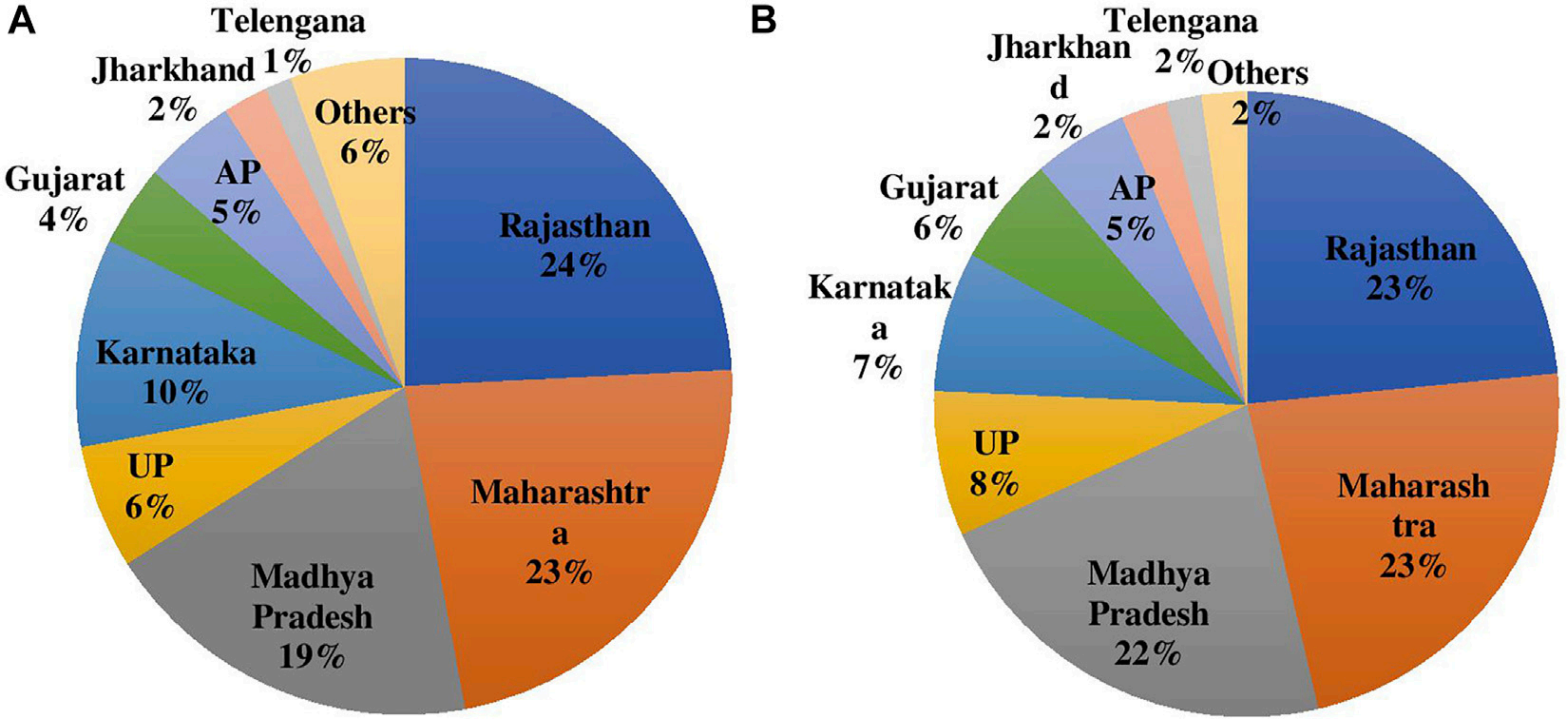
**(A)** Area and **(B)** Production of chickpea during 2020 in major producing States in India.

Rajasthan, Maharashtra, Madhya Pradesh (MP), Uttar Pradesh (UP), Karnataka, and Andhra Pradesh (AP) are the major states growing chickpea and other pulses in India. Rajasthan is also the highest producer of chickpea in India followed by Maharashtra, MP, UP, and Karnataka; and together contribute to 83% of production and 82% of the area in India (Figures 2A,B).

Although the productivity is a little higher than average global productivity, it is lesser than the estimated potential yield, i.e., 6 tones/ha under optimum conditions for the crop (Thudi et al., 2016). Ever-increasing the human population linked with climate change and limited arable land poses a challenge to meet the demands of growing malnutrition and hunger. A lot of efforts had been made by the national and international scientific community to enhance the productivity of chickpeas, but unable to enhance up to a significant level. The reasons underlying are a narrow genetic base and as a result poor genetic gains in the breeding of improved varieties which, leads to the reduction in the yield and its adaptation (Varshney et al., 2012). Devastating pests, pathogens, and increased incidences and severity of abiotic stress amid climate change are the major factors adversely affecting chickpea yield and production. Therefore, diverse sources of variations including wild *Cicer* species need to be explored for the genetic enhancement of chickpeas.

Chickpea performs better in cooler areas since it is a C-3 plant, implying that C-3 plants are better for the winter season. However, the harvest index (HI) in pulses (15%–20%) is low when compared to cereals (45–50%), which is a concerning issue. It is caused by excessive vegetative growth and can be countered by early dry matter partitioning into seeds (Saxena and Johansen, 1990). Despite continued efforts by national and international chickpea improvement programs for the last several decades, the production and productivity of chickpeas have not increased significantly. Probably, this has happened due to the lack of variability for desired plant ideotypes, resistance sources for devastating pests and pathogens, and less responsive behavior of pulses toward modern agricultural practices and inputs. In general, chickpeas and other pulses are grown as a residual or alternative crop in marginal areas, only if the farmers have met their food/income requirements from high productivity- high input responsive crops such as paddy and wheat. After the onset of the green revolution, pulses were further marginalized in their traditional farming systems and local landrace variability in the farmer’s field was lost. Furthermore, chickpea is subjected to various types of biotic and abiotic stresses, which are blamed for much of the crop’s unstable and low yields (Reddy, 2016).

In the production of chickpea, there has been a considerable risk of abiotic stresses. Crop failure is frequently attributed to moisture and temperature stresses, which leave the greatest impact on grain yield. Drought and heat stresses cause forced maturity, resulting in reduced yield. For example, the terminal drought stress in the Mediterranean region when chickpea is grown in the spring season. Drought along with heat stress alone annually reduces productivity by up to 70%. Another major problem in chickpea production is soil salinity and alkalinity. High levels of salinity and alkalinity in both semi-arid tropics and irrigated sections of the Indo-Gangetic plains are a major problem, as most of the pulses are highly sensitive to salinity and alkalinity. Another abiotic factor that limits chickpea grain yield is cold, particularly in temperate regions. Yield is further affected by lack of highly resistant sources in the cultivated gene pool for many of the devastating pathogens and biotic stresses such as dry root rot, ascochyta blight, collar rot, botrytis grey mold (BGM) and *Helicoverpa* species further aggravate the situation (Reddy, 2016). In India, more than 250 insect species have been documented to be harmful to pulses including the chickpea crop.

To achieve higher and stable productivity, it is crucial to breed superior crop varieties with high yield, improved nutrition, disease, and pest resistance to meet the rising global demands. The genetic gains of chickpea and other legume crops are very less as compared to other crops, the reason behind this is the narrow genetic base. To meet the future demand, we have to accelerate genetic gains which are a cyclic process of identifying new variants, carrying selection, and fixing desirable traits. Further, to sustain higher genetic gain for a longer duration, infusion of genetic diversity in modern varieties from landraces and wild *Cicer* species is required. Genomics, high throughput precision phenotyping tools, and artificial intelligence can help in making a desired selection, and in achieving accelerated genetic gain while reducing genetic diversity loss (Varshney et al., 2018).

## 2 Narrow Genetic Base—A Major Bottleneck in Chickpea

Chickpeas have an inherently narrow genetic base as the crop had been subjected to a series of major genetic bottlenecks such as natural selection driven by biotic and abiotic stresses, farmers’ selection pressure (domestication syndrome effect), the introduction of a small set of variability (founder effect), utilization of a very small proportion of variability in the breeding of modern cultivars, etc. (Abbo et al., 2003). Chickpea is a self-fertilization crop, which enhances the probability of loss of variability particularly rare alleles/traits in a population during the selection processes, leading to further narrowing of the chickpea genetic base. Some of the other major factors causing narrowed genetic base of chickpea are areas given below:

• Restricted distribution of wild progenitors of chickpea (*C. reticulatum* is restricted to a small area in SE turkey) (Abbo et al., 2003), which obstructs the gene flow from the wild to the cultivated types.
• Founder effect: similar to any other Neolithic crops, chickpea crop is of monophyletic origin from its wild progenitor and only a limited amount of variability is spread to other parts of the world, causing a genetic bottleneck and narrowed genetic base (Ladizinsky, 1985).
• Domestication syndrome: wild progenitors have ordained to cultivated forms after passing through various genetic modifications and acquiring a combination of traits which might have led to the disappearance of many genes/alleles responsible for input response and higher gain yield (Jain et al., 2014).
• The change from autumn to spring sowing in chickpea: in the Early Bronze Age, the shift of chickpea sowing from autumn to spring to avoid certain biotic stresses, i.e., ascochyta blight. This was possible through the selection for vernalization response in chickpea wild progenitor species; which must have caused a drastic loss of genetic diversity (Abbo et al., 2003).
• The replacement of the land races by elite cultivars produced by modern plant breeding methods which are often developed by genetically similar parental lines and most of the breeding programs shares a limited set of parental lines (Tanksley and McCouch, 1997).

Crop improvement mainly relies on the genetic matter available for exploration through the methods of plant breeding, i.e., classical and molecular breeding. The repeated use of the same germplasm has made very less contribution to the development of the new cultivars. Hence, it could be inferred that chickpea has a narrow genetic base and prompt measures for the transfer of targeted traits from wild *Cicer* species to cultivated one should be taken up by properly evaluating, characterizing, identifying, and utilizing the available germplasm during hybridization programs (Varshney et al., 2021).

In cereals, the amount of yield improvement achieved by breeding is substantially more than chickpea and other pulses. This is probably because the crops have not faced such a harsh bottleneck, and have a comparative broader genetic base (Abbo et al., 2003). The drawback of chickpea breeding programs is their narrow genetic base and unavailability of high input responsive cultivars. In order to develop high-yielding lines, chickpea genetic resources are needed to be explored to broaden the genetic base. Genetic diversity is a major contributor to selection-induced genetic gain, therefore, poor genetic diversity in chickpeas is the major limiting factor in enhancing chickpea yield. As a result, expanding the genetic base of chickpeas is critical for enhancing breeding efficiency. Chickpea wild species are an important genetic resource, especially for biotic and abiotic stress resistance and nutritional quality. Chickpea mutants with novel features like brachytic growing behavior (Gaur et al., 2008), more than three flowers per node–the cymose inflorescence (Gaur and Gour, 2002), determinate (Hegde, 2011), and semi-determinate growth habit (Harshavardhan et al., 2019; Ambika et al., 2021) with the potential to generate futuristic plant types have been identified. In addition, several relevant agro-morphological features and key biotic factors in a variety of wild annual *Cicer* species have been discovered and proposed for their introgressions into the cultivated gene pool to expand the genetic basis (Singh et al., 2014). Therefore, there is an emergent need to strengthen research efforts for identifying useful breeding techniques to enhance the genetic base of chickpea for enhancing genetic gains and finally chickpea yield. One of the greatest challenges in boosting grain legume output is the availability of high-quality seed and other inputs, which is lagging in the chickpea crop and only possible through infusing more and more variability in seed chain systems (David et al., 2002).

## 3 Sources of Genetic Diversity and Broadening of Chickpeagenetic Base

In the past, crop improvement has led to narrowing down of the genetic base resulting in low genetic gains and increased risk of genetic vulnerability. In order to overcome the genetic bottlenecks and create superior gene pools, broadening the genetic base through pre-breeding is required to enhance the utility of germplasm. To attain sustainable growth in chickpeas, new sources of genes need to be identified and incorporated into high-yielding cultivars. The systematic evaluation, characterization, and utilization of wild species-specific targeted genes, to overcome the drawbacks of the abiotic and biotic stresses by broadening the genetic base of chickpea cultivars, are the emergent and immediate requirements. Broadening of the genetic base is now necessary and useful and it is well recognized in all crops mainly in chickpeas and other pulse crops.

The genetic base of cultivated chickpeas is limited (Kumar and Gugita, 2004). Breeders are unwilling to employ exotic germplasm because of linkage drag and/or loss of adaptive gene complex, which necessitates a prolonged time for developing cultivars. As a result, breeders prefer to focus on adapted and improved materials; while ignoring wild relatives, landraces, and exotic germplasm accessible in gene banks (Nass and Paterniani, 2000); thus, further narrowing the genetic base and expanding the gap between available genetic resources and their use in breeding programs (Marshall, 1989). However, substantial diversity among specified parental lines is critical for the success of any breeding program, particularly when the traits to be improved are quantitative, highly variable, and exhibit high G × E interactions.

### 3.1 Sources for Broadening of Genetic Base

There are several sources that could be used for broadening of the genetic base in chickpea to overcome the bottleneck of biotic and abiotic stress in the scenario of changing climatic conditions. Tolerance may be contained in the wild relatives, landraces, advanced breeding materials, initial breeding material, and high-yielding cultivars (Meena et al., 2017). Landraces and wild progenitors have been used for the introgression of various abiotic and biotic stress tolerant gene(s). Mini core germplasm (Upadhyaya et al., 2013) along with several varieties and cultivars have been screened intensively for various biotic and abiotic stresses and used for numerous tolerances in chickpeas.

#### 3.1.1 Sources of Chickpea Genetic Diversity: Cicer Wild Relatives

The genus *Cicer* currently comprises 44 species (Table 1) containing 10 annuals and 34 perennials (van der Maesenet al., 2007). *C. turcicum*is the recent most identified wild *Cicer* species endemic to Southeast Anatolia (Turkey) (Toker et al., 2021). This is an annual species, and with sequence similarity based on the internal transcribed spacer (ITS) region, it appears that *C. turcicum* is a sister species of *C. reticulatum* and *C. echinospermum*, both of which gives fertile progenies when crossed with the cultivated species. Utilization of the new species in the chickpea improvement program will have a great impact on the genetic base broadening. *C. arietinum* is the only species that is extensively recognized as cultivated species. *Cicer reticulatum* is identified as a probable ancestor of chickpea (Ladizinsky and Adler, 1976a). The cultivated chickpea is believed to be originated in the Anatolia of Turkey (Van der Maesen, 1984). Vavilov specified two primary centres of origin for chickpea, southwest Asia and the Mediterranean with the secondary center of origin as Ethiopia. The chickpea closely associated species viz.; *C. bijugum*, *C. echinospermum*, and *C. reticulatum* are widely distributed across southeastern Turkey and neighboring Syria (Ladizinsky and Adler, 1975; Ladizinsky, 1998). However, several *Cicer* species are restricted to particular geographic areas such as *C. bijugum* in Syria and Turkey, *C. anatolicum* in Armenia and Turkey, *C. macracanthum* in Pakistan, *C. microphyllum* in India and Pakistan, and so on. *C. arietinum* is a cultivated species that can’t colonize without human assistance. *C. reticulatum* and *C. bijugum* grow naturally in weedy habitats (fallow lands, road sides, cultivated fields of wheat, and other territories not grabbed by human beings or livestock), *C. pungens* and *C. yamashitae* are found in mountain slopes among rubbles, *C. montbretia* and *C. floribundum* are distributed on forest soils, in broad leaf or pine forests and *C. microphyllum* grows naturally in stony and desert areas of the Himalayas in India (Chandel, 1984). Different *Cicer* species and their distributions are presented in Table 1.

**TABLE 1:** List of Cicer species and their distribution.

Sl. No.	Cicer species	Distribution
	Annuals	
1.	*C. arietinum*	Mediterranean region to Myanmar, Ethiopia, Mexico, Chile
2.	*C. bijugum*	Turkey, Syria, Iraq
3.	*C. chorassanlcum*	Afghanistan, Iran
4.	*C. cuneatum*	Ethiopia, Egypt, Sudan, Saudi Arabia
5.	*C. echinospermum*	Turkey, Anatolia, Iraq
6.	*C. judaicum*	Palestine, Lebanon
7.	*C. pinnatifidum*	Cyprus, Iraq, Syria, Turkey, Armenia
8.	*C. reticulatum*	Turkey
9.	*C. yamashitae*	Afghanistan
10.	*C. turcicum*	Southeast Anatolia (Turkey)
	Perennials	
11.	*C. acanthophyllum*	Afghanistan, Pakistan, Tadzhik SSR
12.	*C. anaiolicum*	Turkey, Iran, Iraq, Armenia
13.	*C. atlanticum*	Morocco
14.	*C. balcaricum*	Caucasus
15.	*C. baldshuanicum*	Tadzhik SSR
16.	*C. canariense*	Canary Islands, Tenerife and La palma
17.	*C. fedtschenkoi*	KirghizSSR, Tadzhik SSR, NE Afghanistan
18.	*C. flexuosum*	KirghizSSR, Tadzhik SSR: Tian-shan
19.	*C. floribundum*	Turkey
20.	*C. graecum*	Greece
21.	*C. grande*	Uzbek SSR, Naratau
22.	*C. heterophyllum*	Turkey
23.	*C. incanwn*	Former USSR
24.	*C. incisum*	Greece, Turkey, Iran, Lebanon, Georgian SSR
25.	*C. isauricum*	Turkey
26.	*C. kermanense*	Iran
27.	*C. Korshinskyi*	Tadzhik SSSR
28	*C. laetum*	Description not traced
29.	*C. macracanthum*	Afghanistan, India, Pakistan, Tadzhik SSR
30.	*C. microphyllum*	Afghanistan, Tibet, India, Pakistan, Pamir USSR
31.	*C. mogoltavicum*	Tadzhik SSR
32.	*C. montbrettii*	Albania, Bulgaria, Turkey
33.	*C. multijugum*	Afghanistan
34.	*C. nuristanicum*	Afghanistan, India, Pakistan
35.	*C. oxyodon*	Iran, Afghanistan, Iraq
36.	*C. paucijugum*	Tadzhik SSR
37.	*C. pungens*	Afghanistan, Former USSR
38.	*C. rassuloviae*	Description not traced
39.	*C. rechingeri*	Afghanistan
40.	*C. songaricum*	Tadzhik SSR, Kazakh SSR
41.	*C. spiroceras*	Iran
42.	*C. stapfianum*	Iran
43.	*C. subaphyllum*	Iran
44.	*C. tragacanthoides*	Iran, Turkmen SSR

**FIGURE 3:**
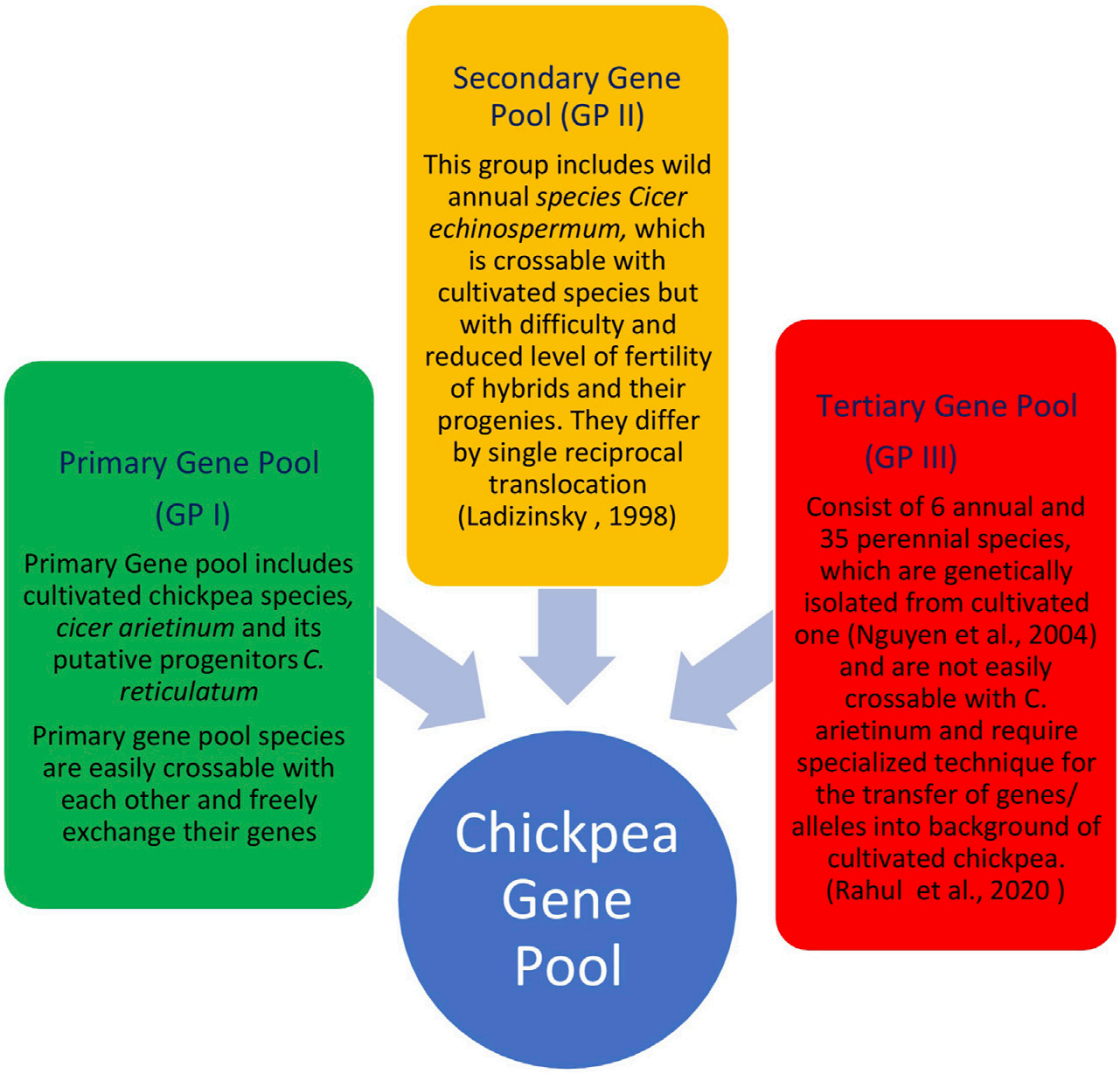
Chickpea gene pool concept and their crossing compatibility.

The primary gene pool constitutes domesticated chickpea, *C. arietinum,* and the immediate progenitor, *C. reticulatum*, the species which are easily crossable with regular gene exchange. They differ either by a reciprocal inversion, a paracentric inversion or by the location of chromosomal satellites (Ladizinsky, 1998). The *C. echinospermum* represents a secondary gene pool and is crossable with cultivated chickpea, but gives reduced pollen fertility in the hybrids and their advanced generations. The tertiary gene pool contained remnant 6 annual and 34 perennial species having poor crossing compatibility with cultivated chickpea and requiring advanced approaches for gene transfer. Wild lines of chickpeas are very good sources of the genes/QTLs for the development of varieties which could be climate-resilient and tolerant to most of the biotic and abiotic stresses (Table 2). These lines consist of different species of chickpea of the primary, secondary, and tertiary gene pool (Figure 3). The resistance transfer from wild species poses several problems such as cross incompatibility, hybrid sterility, hybrid inevitability, and linkage of undesirable traits.

**TABLE 2:** Sources of desirable traits in Cicer species for introgression into elite genetic background of chickpea to broaden genetic base.

S. No.	Trait of interest	Cicer species	References
	Biotic stresses		
1.	Aschochyta blight resistance	*C. arietinum, C. judaicum, C. reticulatum, C. montbretii, C. bijugam, C. pinnnatifidum, C. cuneatum, C. echinospermum*	Vander Maesen and Pundir (1984), Singh and Reddy (1993), Singh et al. (1994), Singh et al. (1998), Collard et al. (2001), Collard et al. (2003), Ahmad et al. (2013), Shah et al. (2005), Pande et al. (2005), Pande et al. (2006), Pande et al. (2010), Kaur et al. (2013), Singh et al. (2014), Benzohra et al. (2014), Li et al. (2017)
2.	Botrytis grey mouldresistance	*C. judaicum, C. bijugam, C. pinnnatifidum, C. reticulatum*	Singh (1982), Van der Maesen and Pundir (1984), Haware et al. (1992), Pande et al. (2006), Basandrai et al. (2006), Basandrai et al. (2008), Kaur et al. (2013), Singh et al. (2014), Hegde et al. (2018)
3.	Cyst nematode resistance	*C. bijugam, C. pinnnatifidum, C. reticulatum*	Greco and Vito (1993), Singh et al. (1994), Ahmad et al. (2013), Singh et al., 2010
4.	Fusarium wilt resistance	*C. arietinum, C. reticulatum, C. bijugam, C. judaicum, C. pinnnatifidum, C. echinospermum, C. cuneatum*	Nene and Haware (1980), Van der Maesen and Pundir (1984), Kaiser et al. (1994), Infantino et al. (1996), Nguyen et al. (2004), Singh et al. (1994), Singh et al. (2005), Ahmad et al. (2013)
5.	Phytophthora root rot resistance	*C. reticulatum, C. bijugum, C. pinnnatifidum, C. Echinospermum*	Knights et al. (2008)
6.	Root-knot nematode resistance	*C. bijugum, C. judaicum, C. pinnnatifidum, C. reticulatum, C. echinospermum*	Singh et al. (2014)
7.	Root-lesion nematode resistance	*C. echinospermum, C. reticulatum*	Thompson et al. (2011)
8.	Rust resistance	*C. bijugam, C. reticulatum, C.echinospermum*	Sillero et al. (2012)
9.	Stem rot resistance	*C. reticulatum, C. pinnatifidum, C. judaicum, C. yamashitae*	Kaur et al. (2008)
10.	Bruchids tolerance	*C. reticulatum*	Singh et al. (2010), Eker et al. (2018)
11.	Helicoverpa pod borer tolerance	*C. bijugum, C. reticulatum, C. echinospermum, C. cuneatum, C. pinnatifidum, C. Microphyllum*	Kaur et al. (1999), Sharma (2004), Sharma et al. (2006), Golla et al. (2018)
12.	Leaf miner tolerance	*C. reticulatum, C. judaicum, C. bijugam, C. cuneatum*	Singh and Weigand (1994), Singh et al. (1994)
13.	Seed beetle tolerance	*C. cuneatum, C. judaicum, C. reticulatum, C. echinospermum*	Gupta and Parihar, (2015)
	Abiotic stress		
14.	Cold tolerance	*C. echinospermum, C.reticulatum, C. bijugum, C. pinnnatifidum, C. judaicum*	Singh et al. (1990), Singh et al. (1995), Sandhu (2004), Toker (2005), Berger et al. (2005), Saeed and Darvishzadeh (2017)
15.	Drought tolerance	*C. anatolicum, C. reticulatum C. microphyllum, C. oxydon, C. montbrettii, C. pinnnatifidium, C. songaricum, C. echinospermum*	Toker et al. (2007), Canci and Toker (2009), Maqbool et al. (2017)
16.	Heat resistance	*C. pinnatifidum, C. reticulatum*	Canci and Toker (2009), Devasirvatham et al. (2012)
17.	Salinity resistance	*C. microphyllum*	Srivastava et al. (2016)
	Yield parameters		
18.	High no. of seeds plant–1	*C. cuneatum, C. montbretii*	Robertson et al. (1995), Robertson et al. (1997), Gupta et al. (2017)
19.	Yield attributes	*C. reticulatum, C. pinnatifidum*	Jaiswal and Singh (1989), Singh et al. (2005), Singh et al. (2012); Singh et al. (2014), Gupta et al. (2017)

#### 3.1.2 Sources of Chickpea Genetic Diversity: Gene Bank Collections and Introductions

The primary goal of a germplasm collection is to capture a significant amount of genetic variation, conserve, and enhance utilization (Singh and Singh, 1997). The first exploration expedition, led by the United States Department of Agriculture’s Regional Pulse Improvement, was conducted in India in the 1970s, collecting almost 7,000 chickpea accessions. In India, systematic explorations to expand chickpea germplasm began only after the establishment of the National Bureau of Plant Genetic Resources (NBPGR) in 1976. In India, the area surveyed for chickpea germplasm collection included regions of Rajasthan, Odisha, Maharashtra, Gujarat, eastern parts of Arunachal Pradesh, Bihar, and southern parts of Tamil Nadu and Karnataka (Singh and Singh, 1997). The awareness about the wild *Cicer* species as rich sources of genes/alleles not just for biotic and abiotic stresses, but also for superior agro-morphological features, has sparked a lot of interest in the researchers (Van der Maesen and Pundir, 1984). Chickpea collection displays variations in plant height, foliage color, pod size, pod bearing habit, seed coat texture, seed coat surface, seed color, and seed size (Singh et al., 2001; Archak et al., 2016). Madhya Pradesh collections were double podded, large-seeded (kabuli type), and tuberculated seeded (desi type) with short and medium duration (Pundir and Reddy, 1989; Pundir et al., 1990). NBPGR has introduced valuable germplasm material from many agroecological zones throughout the world. Some of the potential exotic *Cicer arietinum* germplasm exhibit significant levels of resilience to biotic and abiotic stresses. The imports of *Cicer* wild species (*C. canariense, C. anatolicum, C. oxyodon, C. bijugum, C. reticulatum, C. pinnatifidum* and *C. judaicuni*) have received special attention for use in breeding programs. The majority of the introductions came from International Center for Agricultural Research in the Dry Areas (ICARDA). Other important introduction sources included Spain, Afghanistan, The Former Soviet Union, Iran, United States, Morocco, and Greece. Some of the introduced chickpea lines made significant contributions to the genetic enhancement and pre-breeding, mainly for resistance to Fusarium wilt, Ascochyta blight, leaf miner, cyst nematode, cold, drought, earliness, tall stature, and bold seeds. The important chickpea germplasm collections, including wild species that have been preserved in *ex-situ* collections in various gene banks around the world (Table 3).

**TABLE 3:** *Ex-situ* conservation of Cicer accessions in the world.

Sl. No.	Country	Gene bank name	Cultivated	Wild relatives	Breeding materials	Others	Total number of accessions
1.	Global	International Crop Research Institute for the Semi-Arid Tropics (ICRISAT)	18,842	308	1,317	297	20,764
2.	Global	International Centre for Agricultural Research in Dry Areas (ICARDA)	6,816	547	5,903	2,102	15,368
3.	India	National Bureau of Plant Genetic Resources (NBPGR), New Delhi	14,635	69	—	—	14,704
4.	Australia	Australian Temperate Field Crops Collection (ATFCC)	8,409	246	—	—	8,655
5.	United States	Western Regional Plant Introduction Station, USDA-ARS, Washington State University	7,742	194	102	—	8,038
6.	Iran	National Plant Gene Bank of Iran, Seed and Plant Improvement Institute (NPGBI-SPII)	5,700	—	—	—	5,700
7.	Russia	N.I. Vavilov Research Institute of Plant Industry	1,628	—	558	581	2,767
8.	Pakistan	Plant Genetic Resources Program (PGRP)	2,057	89	—	—	2,146
9.	Turkey	Plant Genetic Resources Department, Aegean Agricultural Research Institute (AARI)	2,047	21	—	7	2,075
10.	Ukraine	Institute of Plant Production nd. a. V. Ya. Yuryev of NAAS	182	24	9	1,542	1,757
11.	Mexico	Estacio´ n de Iguala, Instituto Nacional de InvestigacionesAgrı´colas, Iguala	1,600	—	—	—	1,600
12.	Ethiopia	Institute of Biodiversity Conservation (IBC	1,173	—	—	—	1,173
13.	Hungary	Centre for Plant Diversity	23	5	167	972	1,167
14.	Uzbekistan	Uzbek Research Institute of Plant Industry (UzRIPI)	1,055	—	—	—	1,055
		Total	71,909	1,503	8,056	5,501	86,969

Source:http://www.fao.org/wiews-archive/germplasm_query.htm?i_l¼EN.

#### 3.1.3 Sources of Chickpea Genetic Diversity: Landraces and Cultivated Varieties

Landraces are locally adapted cultivars that evolved in a diverse range of environmental conditions and are maintained generation after generation by farmers and local seed systems. The landraces are the goldmines for trait identification for various biotic and abiotic stresses viz.; drought, salinity and cold. These land races could be exploited in breeding programs for introgression of useful genes/QTLs and enhancing the genetic variability in the modern chickpea cultivars.

The tolerance variation depends on various factors viz.; climatic factors, genotypes, seed attributes, and seed compositions. The most important prerequisite is seedling salinity tolerance since this attribute facilitates the establishment and growth of tolerant genotypes in saline soils. The roles of seed yield, yield components, pods per plant, number of seeds, *in vitro* pollen germination, pollen viability, and *in vivo* pollen tube development to assess the reproductive successful outcome of chickpea under saline stress were investigated (Turner et al., 2013). The increased salt tolerance, as measured under salty ambient by relative yield, was correlated positively with increased shoot biomass, number of pods, and seeds. Pollen viability, *in vitro* pollen germination, and *in vivo* pollen tube growth were uninfluenced by salty ambient in either of the tolerant or sensitive genotypes but pod abortion was relatively higher in salt-sensitive genotypes. Genotypes ICCV-00104, ICCV-06101, CSG-8962, and JG-62 showed a minimum reduction in seedling characters in salt stress conditions. Similar findings were reported by Samineni et al., 2011, while studying chickpea seedlings under saline stress. Flowering terminates at temperatures below 15°C as reported in Australia (Siddique and Sedgley, 1986), India (Savithri et al., 1980; Srinivasan et al., 1999) and the Mediterranean (Singh and Ocampo 1993). It was observed that, when average daily temperature remained below 15°C, plants produced flowers but did not set pods. However, scientists at International Crops Research Institute for the Semi-Arid Tropics (ICRISAT) could develop numerous breeding materials (e.g., ICCV series 88502, 88503, 88506, 88510, and 88516) that are capable to set pods at 12°C–15°C lower average daily temperatures. A pollen selection was applied in Australia to transfer chilling tolerance from ICCV 88516 to chilling sensitive cultivars, leading to the development and release of two chilling tolerant cultivars namely Sonali and Rupali (Clarke and Siddique, 2004). Minicore germplasm was screened for drought tolerance and a few germplasm accessions viz.; ICC series 1356, 3512, 4872, 13523, and 15697 with deeper root systems were identified. The Germplasm accession ICC8261 had the highest root length density, an extremely high root/shoot ratio and rooting depth in both Rabi and Kharif seasons. ICC4958, which is a source used as a deep and large root system parent or check in most drought avoidance studies, was reported to be an extremely prolific rooting genotype. The new genotypes identified could be used as valuable alternative sources for diversification of mapping populations with varying characters and growth durations to obtain the required polymorphism for successfully mapping root traits in chickpeas.

### 3.2 Approaches for Broadening the Genetic Base

**FIGURE 4:**
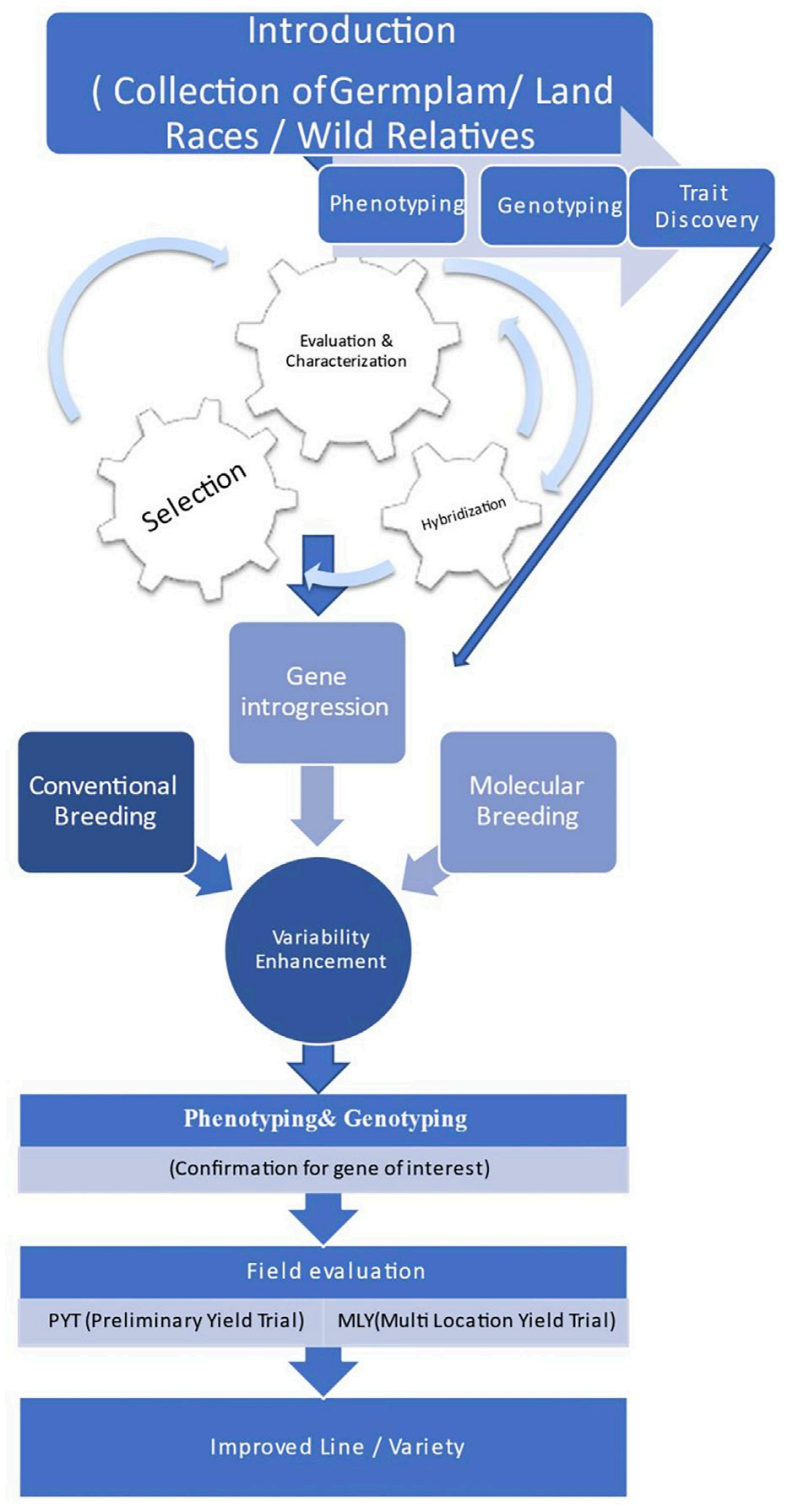
Comprehensive approach for broadening the genetic base of chickpea.

Broadening of the genetic base, up to now, has utilized the techniques of classical breeding viz.; hybridization, segregation, back crossing, cyclic population improvement, pedigree selection among selfed progenies. However, wild relatives couldn’t be utilized because of inter-specific hybridization barriers, limited data for specific traits, and linkage drag. With the advent of molecular breeding techniques, new biotechnological methods, which are being applied for the identification of the QTLs for the traits of interest and needs to be incorporated through various techniques of pre-breeding which are used in transferring useful genes from the exotic or wild species into the high-yielding cultivars. The halted speed of chickpea breeding due to narrow genetic diversity could be fastened by employing wild relatives as a valuable source of new genes and alleles to be further exploited by breeders for allelic richness and broadening of chickpea germplasm. Thus, comprehensive approaches could be utilized for broadening the genetic base in chickpea and other grain legume crops as depicted (Figure 4).

Chickpea’s limited genetic base is a major source of anxiety for chickpea breeding programs, as genetic variability is a major contributor to selection-induced genetic gain. As a result, expanding the genetic base of chickpeas is critical for enhancing breeding efficiency. Chickpea wild species are an important genetic resource, especially for biotic and abiotic stress resistance and nutritional quality. Chickpea mutants with novel features like brachytic growing behavior (Gaur et al., 2008), more than three flowers per node—the cymose inflorescence (Gaur and Gour, 2002), determinate (Hegde, 2011), upright peduncle podding (Singh et al., 2013) and semi-determinate growth habit (Harshavardhan et al., 2019; Ambika et al., 2021) with the potential to generate futuristic plant types have been identified. In addition, several relevant agro-morphological features and key biotic factors in a variety of wild annual *Cicer* species have been discovered and proposed for their introgressions into the cultivated gene pool to expand the genetic basis (Singh et al., 2014). Some of the useful agro-morphological traits including major biotic and abiotic stresses are presented in Tables 2, 4. There is an emergent need to strengthen research efforts for identifying useful breeding techniques to enhance the genetic base of chickpeas.

**TABLE 4:** Sources of resistance to abiotic and biotic stresses as reported by various workers after evaluating the chickpea mini core collection.

Stress	Resistant genotype		References
	Desi	Kabuli	
Drought	ICC- 283, 456, 637, 708, 867, 1205, 1422, 1431, 1882, 2263, 2580, 3325, 4495, 4593, 5613, 5878, 6874, 7441, 8950, 10399, 10945, 11121, 11944, 12155, 12947, 13124, 14402, 14778, 14799, 14815, 15868, 16524	ICC- 4872, 5337, 7272, 7323, 8261, 16796	Kashiwagi et al. (2005), Kashiwagi et al. (2006b), Kashiwagi et al. (2008), Kashiwagi et al. (2010), Parameshwarappa and Salimath (2008), Krishnamurthy et al. (2010), Mulwa et al. (2010), Zaman-Allah et al. (2011a), Zaman-Allah et al. (2011b)
Salinity	ICC- 283, 456, 708, 867, 1431, 2263, 2580, 3325, 4495, 4593, 5613, 5878, 6279, 6874, 7441, 9942, 10399, 10945, 11121, 11944, 12155, 13124, 14402, 14778, 14799, 15868, 16524	ICC- 4872, 7272, 8261, 16796	Serraj et al. (2004), Vadez et al. (2007), Krishnamurthy et al. (2011b)
Heat	ICC- 283, 456, 637, 708, 1205, 1882, 2263, 4495, 5613, 5878, 6874, 7441, 10945, 11121, 11944, 12155, 13124, 14402, 14778, 14799, 14815, 15868	—	Krishnamurthy et al. (2011a), Upadhyaya et al. (2011)
Ascochyta blight	ICC- 1915, 7184, 11284	—	Pande et al. (2006)
Botrytis gray mold	ICC- 2990, 4533, 6279, 7554, 7819, 11284, 12028, 12155, 13219, 13599, 15606, 15610	ICC- 9848, 11764, 12037, 12328, 13816, 14199, 15406	Pande et al. (2006)
Dry root rot	ICC- 1710, 2242	ICC- 2277, 11764, 12328, 13441	Pande et al. (2006)
Fusarium wilt	ICC- 1710, 1915, 2242, 2990, 3325, 4533, 5135, 6279, 6874, 7184, 7554, 7819, 12028, 12155, 13219, 13599, 14402, 14831, 15606, 15610	ICC- 2277, 9848, 12037, 13441, 13816, 14199	Pande et al. (2006)
Pod borer	ICC- 3325, 5135, 6874, 14402, 14831, 15606	ICC- 15406	ICRISAT (2009), Mulwa et al. (2010)
Herbicide	ICC- 2242, 2580, 3325	—	Taran et al. (2010)

#### 3.2.1 Utilization of Adapted and Un-Adapted Germplasm for Traits Discovery and Broadening the Genetic Base

Pre-breeding offers an unparallel opportunity for the introgression of desired genes and gene combinations from exotic germplasm into genetic backgrounds easily employed by breeders with minimal linkage drag (Sharma et al., 2013). Comprehensive broadening of the genetic base through incorporation is the most suitable method when new genetic variabilities for quantitative traits are required, latest and most reliable methods could be optical contribution selection (OCS) based pre-breeding, haplotype-based genomic approaches, and genomic predictions (Varshney et al., 2021). To achieve the highest level of yield, the existing variability among indigenous germplasm has been used. Wild *Cicer* species and exotic germplasm lines include valuable alleles that, if discovered, can aid in breaking yield barriers and improving resistance to various stresses for crop yield stability (Labdi et al., 1996; Tayyar and Waines, 1996; Ahmad and Slinkard, 2003; Ahmad et al., 2005).

Several inter-specific crosses between *Cicer arietinum* and its annual wild relatives have been attempted in the context of wild *Cicer* species usage. There is no evidence of successful hybridization between a perennial *Cicer* species and *Cicer arietinum*. Ladizinsky and Adler (1976b) reported inter-specific crosses amongst *C. arietinum, C. reticulatum* and *C. cuneatum* for the first time. Several researchers have successfully attempted inter-specific hybrids between *Cicer arietinum* and *Cicer echinospermum* (Verma et al., 1990; Singh and Ocampo, 1993; Pundir and Mengesha, 1995). Numerous crossings between *Cicer arietinum* as the female parent and *Cicer reticulatum, C. echinospermum, C. judaicum, C. bijugum*, and *C. pinnatifidum* as the male parent have been conducted (Verma et al., 1990). Van Dorrestein et al., 1998 aimed to cross *C. arietinum* with *C. judaicum* and *C. bijugum*. Badami et al., 1997 used an embryo rescue strategy to successfully hybridize *C. arietinum* with *C. pinnatifidum*. Inter-specific crosses have resulted in the development of certain pre-breeding lines at IIPR, Kanpur, and PAU, Ludhiana (Singh et al., 2012). Singh et al. (2015) attempted inter-specific crosses and the results revealed a high level of heterosis for the number of pods and seed yield per plant in the F_1_ generation. Three cross-combinations viz.; Pusa 1103 x ILWC 46, Pusa 256 x ILWC 46, and Pusa 256 x ILWC 239 demonstrated significantly increased variability for crucial yield related characteristics.

Adoption and harmonizing conventional and modern approaches like molecular breeding, physiological breeding, biotechnological methods, high throughput genomics, and phenomics will aid in the broadening of the genetic base and release of high-yielding varieties which will be tolerant to various biotic and abiotic stresses. Several mapping populations could be developed for the identification of trait-specific QTLs and can be introgressed into high-yielding cultivars for enhancing the gene pool of chickpea.

#### 3.2.2 Bi-Parental Populations for Broadening Genetic Bases

Two inbred lineages are generally crossed in bi-parental populations to generate one or more segregating progenies (Xu et al., 2017). This is the basic approach of combining desired traits in a genotype through ongoing breeding programs. Parents are chosen for a trait of interest based on their genetic and phenotypic diversity allowing the reconstruction of progeny genomes from founder haplotypes to find genomic areas related to the target trait (Dell’Acqua et al., 2015). Bi-parental crosses derived populations capture only a modest impression of the genetic determinants that influence targeted traits in the species and suffer from a lack of diversity owing to the limited genetic base of both parents. Therefore, while the approach is indispensable for any breeding program, genetic diversity must not be reduced in the selection process, to sustain genetic gains for a longer duration. Molecular tools such as re-sequencing technologies and other cost-effective genotyping technologies, which can scan the whole genome, may be useful in the identification of diverse parental lines having the target traits of interest. The utilization of such parental lines will enhance the genetic diversity in the released varieties without compromising the desired yield gain. High-throughput precision phenotyping, genomic selection, and identification of superior haplotypes may further accelerate the breeding cycle and boost the genetic diversity in farmers’ fields to enhance the crop resilience toward the biotic and abiotic stresses. In addition, the QTLs detected in the two-parent population may not be expressed in other genetic origins (Rakshit et al., 2012). Mallikarjuna et al., 2017, utilized F2 populations derived from four crosses (ICCV96029 x CDC frontier, ICC5810 x CDC frontier, BGD 132 x CDC frontier, ICC 16641 x CDC frontier) and found major QTLs corresponding to flowering time genes.

#### 3.2.3 Multi-Parent Populations for Broadening Genetic Bases

Multi-parental and germplasm populations, on the other hand, may offer solutions to bi-parental and germplasm populations’ major flaws. Throughout the history of scientific crop improvement multi-parental populations or multi-parental cross designs (MpCD) have been generated in a range of crop species. Adaptation to crops that are difficult to artificially hybridize, multi-parental populations are created by making crossings amongst more than two inbred founder lines, which serve as a link between association mapping (GWAS) and traditional bi-parental crosses. While such populations are able to combine and reveal better allelic combinations, transgressive segregants, and simultaneously genetic diversity in the progenies are also enhanced. Multi-parent populations also are more efficient in increasing mapping resolution, if they are used for high-density genotyping using advanced high-throughput genomic technologies (Rakshit et al., 2012). This unique technique dramatically improves mapping resolution by merging numerous founder parents with higher phenotypic and genetic diversity. Thanks to the evolution of more powerful techniques, multi-parental populations can now be utilized in numerous genetic mapping studies (Mackay and Powell, 2007; Huang et al., 2015). Here, the emphasis is on MAGIC populations, which are RILs of fine-scale mosaic panels, although numerous MpCD other forms are also available. Thus, MAGIC populations are considered as a growing and next-generation powerful resource for plant genetics mapping, combining variation and high genetic recombination to analyze complex traits’ structure and enhance crop improvement techniques. In various model crop species, MAGIC populations have been generated illustrating their potential to find polymorphisms for underlying QTLs or genes of importance for useful complex traits. There are already MAGIC like or MAGIC populations obtainable in numerous crop species, viz., cereals, legumes, vegetables, fruit trees, and industrial crops with many more in the other works and because of their large genetic foundation, MAGIC populations could be used for discovery of QTL(s) and gene (s), enhancement of breeding populations, introduction and development and of novel genotypes (Pascual et al., 2015). Multi-parent populations such as multiparent advanced generation intercross (MAGIC) populations have gained a tremendous popularity among researchers and breeders. Such populations, along with enhancing genetic diversity, also make it easier to examine the genomic framework and their relationships with phenotypic traits.

#### 3.2.4 Molecular Markers Based Approaches for Broadening Genetic Bases

Since the advent of molecular markers, these tools have played an indispensable role in understanding genetic diversity, phylogenetic relationship, background, and foreground selection in molecular and conventional breeding programs. Recent advances in genomics, coupled with high throughput and precise phenotyping, have made it easier to identify genes that regulate important agronomic attributes. Genetic variability such as multiple podding per peduncle, multiple seeds per pod, upright podding, tall and erect genotypes, and several other traits for biotic stress tolerance are rare, and incorporating these traits to the major cultivars helps in enhancing the variability in the gene pool. These traits could be used in combination with tools for genomics to expedite the generation of crops with higher genetic variability with better agronomic traits, improved resilience to climate change, and nutritional values (Pourkheirandish et al., 2020). Exploring the marker-assisted selection (MAS) technique along with other biotechnological tools can boost genetic diversity and simultaneously enhancing the yield in chickpeas (Varshney et al., 2005; Varshney et al., 2009).

Genomic advancements have aided in understanding the complex trait’s mechanisms affecting chickpeas economically important characters’ genetic architecture as well as productivity in order to speed up breeding programs (Roorkiwal et al., 2020). In chickpea, a number of markers and trait relationships and dense genetic maps have allowed MAS to become a routine practice in crop breeding programs (Kulwal et al., 2011; Madrid et al., 2013; Ali et al., 2016; Caballo et al., 2019). Single nucleotide polymorphism (SNP) allelic variants on 27 ortholog candidate genes were utilized for the GWAS study, and potential candidate genes such as *PIN1*, *TB1*, *BA1/LAX1*, *GRAS8*, and *MAX2* were identified for branch number in chickpea utilizing highly diverse chickpea germplasm (Bajaj et al., 2016). The gene for double podding per peduncle was linked to Tr44 and Tr35 on linkage group 6 (Cho et al., 2002). Saxena et al. (2014) has mapped four traits viz. 100-seed weight, pod, number of branches per plant and plant hairiness, using simple sequence repeats (SSRs) and SNP markers. There are several other examples of utilization of molecular makers for the identification of traits and underlying genes/QTLs in chickpea such as 100-seed weight (Das et al., 2015; Kujur et al., 2015b), resistance to *Helicoverpa armigera* (Sharma et al., 2005), pod number (Das et al., 2016), flowering time (Srivastava et al., 2017), plant height (Parida et al., 2017), photosynthetic efficiency traits (Basu et al., 2019), etc. Furthermore, comprehending the chickpea developmental processes’ regulations has been facilitated by the framework offered due to discoveries of new microRNAs (miRNAs) and their expression patterns (Jain et al., 2014).

For genomic investigations and crop improvement, numerous polymorphic molecular markers that could be exposed to high-throughput analysis are sought. On the basis of isozyme analysis, *Cicer arietinum* is most closely related to *C. reticulatum*, followed by *C. echinospermum, C. bijugum, C. pinnatifidum, C. judaicum, C. chorassanicum, C. yamashitae* and *C. cuneatum* (Ahmad et al., 1992). *Cicer reticulatum* and *Cicer echinospermum* were grouped together in the same cluster; *Cicer chorassanicum* and *Cicer yamashitae* were grouped together in another cluster; *Cicer bijugum*, *Cicer judaicum*, and *Cicer pinnatifidum* were grouped together in the third different cluster; and *Cicer cuneatum*alone formed the fourth different cluster based on the analysis of RAPD markers (Ahmad, 1999; Sudupak et al., 2002). An AFLP analysis for the same *Cicer* species also confirmed the same pattern (Sudupak et al., 2004). RAPD and ISSR fingerprinting demonstrate that *C. arietinum* cultivars had the narrowest genetic variation while its wild *C. reticulatum* accessions had much greater genetic variation, which could be used in chickpea improvement (Rao et al., 2007). The widespread use of molecular markers in chickpea genetics and breeding began with the introduction of SSR markers. The draft genome sequence of chickpea identified approximately 48,000 SSRs appropriate for PCR primer design for use as genetic markers (Varshney et al., 2013), whereas a draft sequence of *C. reticulatum* (PI 4889777) spanning 327.07 Mb was assembled to the eight linkage groups with 25,680 protein-coding genes (Gupta et al., 2017).

A variety of comparatively new marker systems have recently been introduced including sequence-based SNP and hybridization-based diversity array technology (DArT) markers which offer medium to high-throughput genotyping and are simple to automate. Two sets of Axiom®CicerSNP array have been developed in chickpea, one was including 50,590 probes distributed on all eight linkage groups as described by Roorkiwal et al. (2014) and the second multispecies SNP chip includes chickpea along with other pulses using markers that can be imputed up to whole-genome (800,000 markers) was developed by AgriBio, Centre for AgriBioscience Melbourne, Australia (personal communication).

To date, several studies have been published using DArT and SNP chips. We highlight the 5397 polymorphic DArT markers identified from a pool of 15,360 developed markers utilizing 94 different chickpea genotypes (Thudi et al., 2011). The low genetic diversity was unraveled between wild *Cicer* and cultivated species through DArT markers (Roorkiwal et al., 2014). Although transcriptome investigation of chickpea and its wild progenitors detected thousands of SNPs (Coram and Pang, 2005; Varshney et al., 2009; Gujaria et al., 2011; Agarwal et al., 2012; Bajaj et al., 2015b; Kujur et al., 2015a). These SNPs and markers can be utilized by chickpea breeders in MAS-assisted breeding programs.

#### 3.2.5 Trait Identificationin Legumes for Broadening Genetic Bases

##### 3.2.5.1 Trait Identification Through Sequencing

With the advancement in the next-generation sequencing (NGS)-based approaches, trait mapping has become an easy job to do. Not only are these technologies time-saving but also cutting the cost at basal levels. The genetic mapping is based on recombination (the exchange of DNA sequence between sister chromatids during meiosis) and the distance between the markers measured by cM representing approximately 1% of the recombination frequency, while the physical map is based on the alignment of the DNA sequences, with the distance between markers measured in base pairs. However, the high-resolution physical maps serve as the scaffold for genome sequence assembly to identify the most accurate distance between the markers and the genes linked in addition to exploring the potential candidate gene(s) linked to desired traits. The trait mapping through sequencing approaches may be categorized into two classes 1) Sequencing of complete populations for trait mapping and 2) Sequencing of pooled samples for trait mapping. Using composite interval mapping a high-density genetic map consisting of 788 SNP markers spanning through 1125cMalong with the identification of 77 QTLs for 12 traits was reported (Jha et al., 2021). Similarly, several QTLs were mapped for several other traits like flowering time (Mallikarjuna et al., 2017; Jha et al., 2021), plant height (Kujur et al., 2016; Barmukh et al., 2021), and primary branches (Barmukh et al., 2021).

##### 3.2.5.2 Trait Identification Through Sequencing of Complete Populations

It primarily consists of the genotyping by sequencing (GBS) and whole-genome re-sequencing (WGRS) mapping populations, both of which yield genome-wide SNPs. GBS is popular because it is inexpensive and provides a lot of genetic data. The discovery of a large number of genome-wide SNPs has facilitated rapid diversity assessment, trait mapping, GS and GWAS in a variety of crop by employing GBS—a potential strategy. A chickpea genetic variation map was developed using whole-genome sequencing technique and genomes were characterized at the sequence level, observing variations in 3,171 cultivated and 195 wild accessions and construction of a pan-genome to explain the genomic diversity across wild progenitors and cultivated chickpea (Varsheny et al., 2021). The 16 mapping populations segregating for different abiotic (drought, heat, salinity), biotic stress (Fusarium wilt, Aschochyta blight, BGM & *Helicoverpa armigera*) and protein contents along with their 35 chickpea parental genotypes were re-sequenced in order to exploit the genetic potential for chickpea improvement (Thudi et al., 2016). Genetic analysis, fine-tuning of genomic areas, and production of genetic maps are facilitated by re-sequencing (Kujur et al., 2015b; Li et al., 2015). Chickpea is one of the best examples of crops in which GBS was used to identify 828 SNPs in addition to the previously mapped SSRs. The creation of these detailed genetic maps aids in the discovery of QTLs in chickpea that controls yield, drought tolerance, and seed weight. It is quite useful for locating QTL hotspots. Moving on to the second promising strategy, WGRS has been found to be more useful in finding candidate genes than GWAS (Jaganathan et al., 2015; Varshney et al., 2014).

##### 3.2.5.3 Trait Identification Through Pooled Sequencing

The analysis is done on the basis of the pooled population through the inclusion of BSR-Seq, Indel-Seq, Mut-Map, QTL-Seq, and Seq-BSA the five major approaches. The “QTL-Seq” is the first and foremost promising technique to have been successfully employed with larger crop plant genomes. This strategy has been used to pinpoint the blast resistance and seedling vigor governing genomic areas in rice, flowering QTLs in cucumber, fruit weight and locule number loci in tomatoes and successfully applied for localization of QTLs/candidate genes for 100 seed weight in chickpea (Takagi et al., 2013; Li et al., 2015). The “MutMap” is a robust and simple NGS-based approach, first of all which was applied for the identification of EMS-induced interesting candidate genes in rice. Crossing of selected mutant plants with wild types, which reduces background noise—the fundamental benefit, is the necessity of mapping the population created for the MutMap experimental strategies. Consequently, using extreme pool samples derived from segregating populations coupled to a wild parent the genome-wide SNP index is calculated. The third method, known as “Seq-BSA,” is a straightforward and reliable NGS-based strategy for identifying potential SNPs in specific genomic regions (Takagi et al., 2013). Employing QTL-seq pipelines utilizing parent with high-value trait as reference parent assemblage, genome-wide SNP indexes of both extreme bulks are calculated in the third method. The fourth strategy, “Indel-Seq” which is mostly focused on insertions and deletions, has also emerged as a potential trait mapping approach. To date, the proposed methodologies for identifying genomic regions have relied on the discovery of SNPs followed by the use of various statistical approaches to recognize candidate genomic gene/regions. However, in all approaches, the relevant genomic region-specific existing Indels have not been targeted for trait mapping but ignored. The fact that the Indels reported in the candidate genes are found in most of the cloned genes in rice and other crops and makes this strategy more practicable. The strength of the RNA-seq and BSA were combined for enhancing the strength to find candidate genes for the targeted characteristic—a novel genetic mapping approach as the fifth strategy, dubbed as “Bulked segregant RNA-Seq (BSR-Seq)”. This method has been used to successfully identify the glossy3 genes in maize. RNA-seq-based investigations will be cheaper than WGRS at higher coverage; hence, this strategy has more cost savings. We believe that, given the benefits of RNA-Seq, this approach will be effective for legumes with larger genomes (Liu et al., 2012; Trick et al., 2012). Thus, chickpea breeders utilize these generated informations in chickpea MAS-assisted breeding programs.

#### 3.2.6 Transcriptomics Utilization for Broadening the Genetic Bases

Work on legumes focused on building libraries of cDNAs, gene expression profiling, the manufacture of expressed sequence tags (EST), and *in silico* extraction of EST data sets’ functional information even before sequences of the genome achievability. Transcriptome sequencing has been employed in other functional genomics methodologies, viz., genome annotation, gene expression profiling, and non-coding RNA identification employed transcriptome sequencing (Morozova and Marra, 2008). In recent years, for generating a large number of transcript reads from a variety of developing and distress-responsive tissues in several leguminous crops through several low-cost sequencing systems has already been established, viz., an improved transcriptome assembly, utilizing FLX/454 sequencing together with Sanger ESTs comprised 103,215 Transcript Assembly Contigs (TACs) with an average contig length of 459 base pairs in chickpea (Hiremath et al., 2011). Employing various sequencing technologies or a combination of two or more sequencing technologies created by transcriptome assemblies provides useful transcriptomic resources such as functional markers, EST-SSRs, Spanning Regions (ISRs), SNPs, Introns, and so on in soybean and common bean 1,682 and 4,099 SNPs, respectively (Deschamps and Campbell, 2012), ESTs comprising of 103,215 Transcript Assembly Contigs (TACs) in chickpea (Hiremath et al., 2011) can be utilized by the breeders to achieve a better grasping of the molecular underpinnings of distress tolerance and as a result more stress-tolerant beans as well chickpea cultivars may be produced and narrow genetic base may be broadened.

#### 3.2.7 Proteomics and Metabolomics for Broadening the Genetic Bases

New datasets for crop plants can be created by exploiting the opportunities of advancement in “omics” technologies. The advancements will result in a greater integrated association of “omics” data and crop improvement resulting in the evolution from genomic assisted breeding (GAB) to omics assisted breeding (OAB) in the future (Langridge and Fleury, 2011) that can also be utilized for broadening the genetic bases in chickpea.

##### 3.2.7.1 Proteomics Approaches

Increased proteome coverage and advancements in quantitative evaluations have benefitted plant proteome composition, modulation, and alterations of developmental phases including stress–response mechanisms. Proteomic pipelines are rapidly being used in crop research notably to investigate crop-specific features and stress response mechanisms. Proteome mapping, comparative proteomics, discovery of post-translational modifications (PTMs), and protein–protein interaction networks are key topics of plant proteomics (Vanderschuren et al., 2013). In chickpea the comparative root proteomic analysis for the effect of drought and its tolerance in hydroponics using 2D gel electrophoresis coupled with MALDI-TOF revealed eight categories of protein-based on their functional annotation viz.; proteins involved in carbon and energy metabolism, proteins involved in stress response, ROS metabolism, signal transduction, secondary metabolism, nitrogen and amino acid metabolism (Gupta and Laxman, 2020). High-throughput protein quantification has benefited from advancements in accuracy, speed, mass spectrometry (MS) utilizations in terms of sensitivity, and software tools. Gel-based or gel-free, shot-gun, and label-based (isotopic/isobaric) or label-free quantitative proteomics platforms have emerged as a result of developments in MS technology for high-throughput protein quantifications (Abdallah et al., 2012; Hu et al., 2015). In legume crops, comparative proteomics approaches and differential expression analyses have given understanding of distress responses including dehydration, and early phases of cold stress in chickpeas (Pandey et al., 2008) and can be effectively integrated into genomic-assisted breeding programs for broadening the narrow genetic bases.

##### 3.2.7.2 Metabolomics Approaches

In plant metabolic engineering, targeted reverse genetic methods and high-throughput metabolite screening have the advantage of providing a better understanding of metabolic networks on a larger scale in relation to developmental stages of phenotypes and the ability to screen out undesirable traits (Fernie and Schauer, 2009). The literature describes two major metabolomics profiling methodologies that use nuclear magnetic resonance (NMR) and MS. A combination of many analytical techniques generated from one of the MS was frequently used to obtain a larger range of numerous metabolites in plants (Arbona et al., 2013). Flow injection-based analysis with Fourier Transform Infrared spectroscopy and MS (FIA/MS) are two further approaches. The identification of new metabolic QTLs and candidates for the desired traits are made possible by combining metabolomics data, transcriptomics data, high-throughput phenotypes, and bioinformatics platforms to profile large genetically varied populations and increase the accuracy of targeted gene identification. To boost yields and broaden the narrow genetic bases, metabolomics is utilized in conjunction with a genomic-assisted selection and introgression techniques, minimizing the time spent in uncovering new characteristics and allelic mutations (Fernie and Schauer, 2009).

#### 3.2.8 Pan Genomics

Recent developments in genome sequencing technologies have revolutionized the crop improvement programs. Now the whole-genome sequencing (WGS) is not limited to one or two individuals, but a large set of accessions of a species (pangenome) including their crop wild relatives (super-pangenome) are the whole genome sequenced to unravel the full potential of the species for the crop improvement programs. Once the pangenome information is available, the genomic segments/genes lacking in cultivated germplasm can be identified and introgressed in cultivated germplasm to enhance the genetic variability. The total number of genes of a species are collectively known as its pan-genome. It was observed from several evidences that a sole organism can’t contain all the genes of a species due to variability present in the genomic sequences. The desirable features of an ideal pan-genome are completeness (i.e., contains all functional genes), stability (i.e., unique catechistic features), comprehensibility (i.e., contains all the genomic information of all the species or individuals), and efficacy (i.e., organized data structure). Pangenome information of a species helps in the identification of desired alleles, rare alleles, presence or absence of a traits in a species. Recently a chickpea pangenome of 592.58 Mb was constructed which containsa total of 29,870 genes (Varshney et al., 2021). The pan-genome was constructed using whole-genome sequencing using 3,366 comprising 3,171 cultivated and 195 wild accessions. Assembly was done by combining the CDC frontier reference genome including 53.60 Mb from cultivated chickpea inclusive of 2.93 Mb from ICC 4958 and 5.28 Mb from 28 accessions of *C. reticulatum*. This pan-genome analysis revealed useful information on genomic regions more often selected during the domestication process, superior haplotypes, and targets for purging deleterious alleles. The new genes identified encoding responses to oxidative stress, response to stimuli, heat shock proteins, cellular response to acidic pH, and response to cold, which could have a possible contribution to the adaptation of chickpea.

#### 3.2.9 QTL Mappings, Their Introgression and Utilization for Broadening the Genetic Bases

The utility of the fundamental assumption of locus finding by co-segregation of characteristics with markers is enhanced by new permutations of QTL mapping (Table 5). However, the definition of a trait can now be expanded beyond whole-organism phenotypes to include phenotypes like the amount of RNA transcript or protein produced by a specific gene because these phenotypes have more typical organismal characteristics viz.; yield in corn are polygenic and QTL mapping works in these situations. Transcript abundance is regulated not only by cis-acting regions like the promoter but also by Transacting transcription factors that may or may not be related. Similarly, local variation at the coding gene and distant variation mapping to other areas of the genome control protein abundance. Local variation is most likely made up of cis variations that regulate transcript levels. Polymorphisms for the protein’s stability or control could be another local mechanism. Distant variation, on the other hand, could comprise upstream regulatory control areas (Upadhyaya et al., 2016).

**TABLE 5:** List of QTLs for various traits in chickpea.

S. No.	Trait	Linkage group	QTL	Position	Reference
Phenological traits					
1.	Plant height	*LG01*	*qPH1.1*	56.984–57.223	Kujur et al., 2016
2.	Plant height	*LG02*	*qPH2.1*	24.496–29.852	
3.	Plant height	*LG03*	*qPH3.1*	26.358–26.536	
4.	Plant height	*LG04*	*qPH4.1*	28.738–28.796	
5.	Plant height	*LG07*	*qPH7.1*	28.738–28.796	
6.	Plant height	*LG08*	*qPH8.1*	44.194–44.882	
7.	Plant height	*LG04*	*qPLHT4.1*	216.23–223.07	
8.	Plant height	*LG01*	*qPLHT1.1*	12.70–13.40	Barmukh et al., 2021
9.	Plant height	*LG04*	*qPLHT4.1*	216.23–223.07	
10.	Plant height	*LG05*	*qPLHT5.1*	1.07–7.62	
11.	Plant height	*LG08*	*qPLHT8.1*	13.74–14.30	
12.	No of primary branches	*LG02*	*qPB.2.1*	111.10–111-40	
13.	No of primary branches	*LG03*	*qPB.3.1*	14.3014.40	
14.	Flowering time	*LG03*	*Qefl1-*1	0.00	Mallikarjuna et al., 2017
15.	Flowering time	*LG04*	*Qefl1-*2	41.00	
16.	Flowering time	*LG01*	*Qefl2-*1	15.00	
17.	Flowering time	*LG03*	*Qefl2-*2	21.00	
18.	Flowering time	*LG04*	*Qefl2-*2	55.00	
19.	Flowering time	*LG08*	*Qefl1-*3	15.00	
20.	Flowering time	*LG03*	*Qefl2-*4	5.00	
21.	Flowering time	*LG03*	*Qefl3-*1	31.00	
22.	Flowering time	*LG08*	*Qefl3-*2	2.00	
23.	Flowering time	*LG06*	*Qefl4-*1	9.00	
24.	Days to flowering initiation (DFI)	*LG06*	*CaDFI_LS6.1*	37.11	Jha et al., 2021
25.	DFI	*LG08*	*CaDFI_LS8.1*	42.71	
26.	DFI	*LG06*	*CaDFI_LS6.1*	37.11	
27.	DFI	*LG08*	*CaDFI_LS6.1*	42.71	
28.	Days to maturity (DM)	*LG01*	*CaDMI_LS1.1*	7.11	
29.	DM	*LG01*	*CaDMI_LS1.2*	152.61	
30.	DM	*LG01*	*CaDMI_LS1.3*	154.81	
Yield and related traits					
31.	Days to pod initiation (DPI)	*LG07*	*CaDPI_LS7.2*	98.01	Jha et al., 2021
32.	DPI	*LG07*	*CaDPI_LS7.1*	97.01	
33.	DPI	*LG06*	*CaDPI_LS6.1*	37.11	
34.	DPI	*LG06*	*CaDPI_LS6.1*	37.11	
35.	DPI	*LG06*	*CaDPI_LS6.1*	37.11	
36.	DPI	*LG01*	*CaDPI_LS1.1*	153.61	
37.	Days to pod filling (DPF)	*LG08*	*CaDPF_LS8.1*	67.41	Jha et al., 2021
38.	DPF	*LG04*	*CaDPF_NS4.2*	136.61	
39.	DPF	*LG04*	*CaDPF_NS4.1*	138.11	
40.	No of filled pods (FP)	*LG06*	*CaFP_NS6.1*	141.40	
41.	100 seed weight (g)	*LG06*	*Ca100SW_LS7.1*	97.01	Jha et al., 2021
42.	100 seed weight (g)	*LG01*	*Ca100SW_LS1.1*	46.21	
43.	100 seed weight (g)	*LG04*	*Ca100SW_LS4.1*	159.71	
44.	100 seed weight (g)	*LG07*	*Ca100SW_LS7.1*	97.01	
45.	100 seed weight (g)	*LG06*	Q100SW6.1	43.66–43.70	Barmukh et al., 2021
46.	100 seed weight (g)	*LG07*	Q100SW7.1	47.61–47.77	
47.	100 seed weight (g)	*LG03*	Q100SW3.1	153.40–167.6	
48.	100 seed weight (g)	*LG06*	Q100SW6.2	87.91–88.02	
49.	100 seed weight (g)	*LG07*	Q100SW7.2	139.78–140.04	
50.	100 seed weight (g)	*LG04*	Q100SW4.1	216.23–223.07	
51.	Seed yield/plant (g)	*LG02*	*CaSYPP_LS2.1*	22.51	Jha et al., 2021
52.	Seed yield/plant (g)	*LG06*	*CaSYPP_LS6.1*	12.21	
53.	Seed yield/plant (g)	*LG06*	*CaSYPP_NS6.2*	52.31	
54.	Seed yield/plant (g)	*LG06*	*CaSYPP_NS6.3*	53.01	
55.	Seed yield/plant (g)	*LG04*	qYPP4.1	86.44–87.52	Barmukh et al., 2021
56.	Seed yield/plant (g)	*LG01*	qYPP1.1	15.00–46.80	
57.	Pods per plant	LG06	qPPP6.1	0.75–1.27	Barmukh et al., 2021
58.	Biological yield/plant		CaBYPP_NS6.1	52.31	Jha et al., 2021
59.	Biological yield/plant	*LG06*	CaBYPP_NS6.1	52.31	
60.	Biological yield/plant	*LG06*	CaBYPP_LS6.3	114.01	
61.	Biological yield/plant	*LG02*	CaBYPP_LS2,1	55.91	
62.	Biological yield/plant	*LG06*	CaBYPP_LS6.4	115.01	
63.	Biological yield/plant	*LG06*	CaBYPP_LS6.5	115.31	
64.	Biological yield/plant	*LG06*	CaBYPP_NS6.2	58.71	
65.	Harvest index (HI %)	*LG05*	CaHI_NS5.1	42.11	
66.	HI %	*LG07*	CaHl_NS7.1	35.81	
67.	HI %	*LG06*	CaBYPP_NS6.3	170.81	
68.	HI %	*LG06*	CaHl_LS6.2	100.21	
69.	HI %	*LG08*	CaHl_LS8.1	43.11	
70.	HI %	*LG07*	CaHl_NS7.2	142.71	
71.	HI %	*LG06*	CaHI_NS6.1	84.21	
72.	HI %	*LG07*	CaHl_NS7.1	35.81	
Physiological traits					
73.	Chlorophyll Content (CHL, ng/mm^2^)	*LG04*	CaCHL_NS4.3	151.51	
74.		*LG04*	CaCHL_NS4.3	151.51	
75.		*LG02*	CaCHL_LS2.1	38.31	
76.	CHL, ng/mm^2^	*LG05*	CaCHL_LS5. 1	44.01	
77.	CHL, ng/mm^2^	*LG05*	CaCHL_LS5.2	44.31	
78.	CHL, ng/mm^2^	*LG04*	CaCHL_NS4.1	142.91	
79.	CHL, ng/mm^2^	*LG04*	CaCHL_NS4.2	150.11	
80.	Cell membrane stability (CMS %)	*LG04*	CaCMS_NS4.1	133.61	Jha et al., 2021
81.	CMS %	*LG06*	CaCMS_LS6.1	67.21	
82.	CMS %	*LG03*	CaCMS_NB3.1	0.01	
83.	Nitrogen balance index (NBI)	*LG08*	CaNBl_LS8.3	3.81	Jha et al., 2021
84.	NBI	*LG08*	CaNBl_LS8.1	0.01	
85.	NBI	*LG08*	CcNBI_LS8.2	1.01	
86.	NBI	*LG07*	CaNBI_LS7.2	97.01	
87.	NBI	*LG07*	CaNBI_LS7.1	34.61	
88.	NBI	*LG08*	CaNBI_LS8.2	1.01	
89.	NBI	*LG06*	CaNBI_LS6.1	69.71	
90.	NBI	*LG08*	CaNBI_LS8. 1	0.01	
91.	NBI	*LG06*	CaNBI_LS6.2	70.71	
92.	NBI	*LG07*	CaNBI_LS7.1	34.61	
93.	Normalized difference vegetation index (NDVI)	*LG02*	CaNDVI_LS2.2	66.01	Jha et al., 2021
94.	NDVI	*LG04*	CaNDVI_NS4.1	68.31	
95.	NDVI	*LG04*	CaNDVI_NS4.2	69.21	
96.	NDVI	*LG02*	CaNDVI_LS2.1	65.41	
97.	NDVI	*LG02*	CaNDVI_LS2.2	66.01	
98.	NDVI	*LG03*	CaNDVI_NS3.1	48.41	
99.	NDVI	*LG08*	CaNDVI_NS8.1	18.61	
100.	NDVI	*LG08*	CaNDVl_NS8.2	18.91	
101.	NDVI	*LG03*	CaNDVI_NS3.1	48.41	
102.	NDVI	*LG06*	CaNDVI_NS6.1	20.01	
103.	NDVI	*LG01*	CaNDVI_LS1.2	44.21	
104.	NDVI	*LG01*	CaNDVI_LS1.1	42.21	
105.	NDVI	*LG05*	CaNDVI_NS5.2	36.11	
106.	NDVI	*LG05*	CaNDVI_NS5.1	35.11	
107.	NDVI	*LG04*	CaNDVI_NS4.1	68.31	

Quantitative trait loci (QTLs) conferring resistance to biotic and abiotic stresses have been applied in chickpeas in the last 2 decades and the molecular markers closely associated with these loci are also located (Santra et al., 2000). For example, several QTLs conferring Ascochyta blight resistance are identified, and several MAS (SCY17 and SCAE19) were reported as the best markers linked to AB-resistant genes. These two markers were validated on different populations (Iruela et al., 2006; Imtiaz et al., 2008; Madrid et al., 2014). More recently, three major conserved quantitative trait loci (QTLs) that confer AB resistance have been reported, two on chromosome Ca2 and one on chromosome Ca4. These QTLs explained a maximum of 18.5%, and 25% of the total variation. In total, 27 predicted genes were located in chromosome IV close to these QTL (Hamwieh et al., Unpublished data).

The 20 QTLs and candidate genes associated with seed traits were also identified in chickpeas using the GBS approach (Pavan et al., 2017). In pigeon pea, the GBS-based mapping of two RIL populations led to the identification of QTLs and candidate genes for resistance to fusarium wilt (FW) and sterility mosaic disease (SMD) (Saxena et al., 2017) in addition to restoration of fertility (Rf) (Saxena et al., 2018), using GWAS drought tolerance-related traits in chickpea (Kale et al., 2015), flowering time control, seed development and pod dehiscence in pigeon pea (Varshney et al., 2017) have been mapped. The GBS has been utilized in the fine mapping of the “*QTL-hotspot*” region for drought tolerance-related traits in chickpeas (Kale et al., 2015). In the case of chickpea, QTL seq approach has successfully identified a major genomic region (836,859–872,247 bp) on Ca1 chromosome which was further narrowed down to a 35-kb region harboring six candidate genes for 100 seed weight (Das et al., 2015).

Plant breeding can help in solving the global problem of micronutrient deficiencies in a cost-effective and long-term manner. The development of biofortified chickpea varieties is aided by evaluating cultivars for micronutrient contents and identifying quantitative trait loci (QTLs)/genes and markers. The F_2:3_ derived population resulting from a cross between MNK-1 and Annigeri-1 was dissected employing the GBS technique and concentrations of Fe and Zn were examined with the goal of determining the responsible genetic areas (Vandemark et al., 2018). The researchers mapped 839 SNPs on an intra-specific genetic linkage map covering a total distance of 1,088.04 cM with a marker density of 1.30 cM. By combining linkage map data with phenotypic data from the F2:3 populations a total of 11 QTLs for seed Fe concentration on CaLG03, CaLG04, and CaLG05 with phenotypic variance varying from 7.2% (CaqFe3.4) to 13.4% (CaqFe3.4; CaqFe4.2). On CaLG04, CaLG05, and CaLG08 along with eight QTLs for seed Zn concentration with explained phenotypic variances ranging from 5.7% (CaqZn8.1) to 13.7% (CaqZn4.3) were discovered (Pandey et al., 2016).

The identification of marker-trait association between a genetic marker and a trait of interest is the initial stride in crop breeding utilizing molecular breeding/genomics assisted breeding. For initial experiments, linkage maps were created employing F_2_ populations. The inter-specific cross *C. arietinum* (ICC 4958) x *C. reticulatum* (PI 489777) was employed to create the first recombinant inbred lines (RILS) mapping population which is now being used as a chickpea reference mapping population for genome mapping (Nayak et al., 2010). Maps created from intra-specific mapping populations have a smaller number of markers (<250 markers) and poorer genome coverage (<800 cM) due to minimal variation in the cultivated chickpea. Consensus genetic maps were also created utilizing both inter and intra-specific mapping populations.

The genetic mapping of QTLs affecting resistance to various diseases, and also vital agronomical traits, in chickpea are extensively documented. Santra et al. (2000) identified two quantitative trait loci (QTL1 and QTL2) that give resistance to Ascochyta blight. These QTLs were predicted to be responsible for overall phenotypic variance (34.4%, 14.6%), respectively (Santra et al., 2000; Tekeoglu et al., 2002). Comparative protein profiling of wild chickpeas and induced mutants was carried out in order to measure genetic diversity between mutants and parental genotypes (Patil and Kamble, 2014). Kujur et al. (2016) reported candidate genes and natural allelic variations for QTLs determining plant height, which was followed by the discovery of QTLs for heat distress response (Paul et al., 2018) as well as photosynthetic efficiency attributes for boosting seed yield in chickpea using GWAS and expression profiling (Basu et al., 2019). These discoveries have opened up new paths for analysis and comprehensive characterization of wild *Cicer* species, which will help in harnessing unidentified allelic variations to extend the genetic foundation of cultivars.

Molecular markers have been discovered for gene(s)/QTL(s) linked to abiotic stress resistances, viz., drought tolerance (Molina et al., 2008; Rehman et al., 2012), salinity resilience (Vadez et al.,2012), biotic stresses, viz., Ascochyta blight (Milla´n et al., 2003; Iruela et al., 2006; Aryamanesh et al., 2010; Garg et al., 2019), Fusarium wilt (Cobos et al., 2005; Gowda et al., 2009; Sabbavarapu et al., 2013) and botrytis gray mold (Anuradha et al., 2011) along with seed characteristics (Gowda et al., 2009) in chickpea. These technologies can be employed to improve chickpea genetics and breeding as well as to explain the variety of the chickpea genome and domestication events. Furthermore, genomic selection has been presented as a promising strategy for enhancing traits that are influenced by a large number of gene (s)/QTL (s) (Bajaj et al., 2015a; Bajaj et al., 2015b). Both phenotypic and genotypic data sets are employed in this approach to determine genomic estimated breeding values (GEBV) of improved progenies.

#### 3.2.10 Genome-Wide Association Studies for Broadening the Genetic Bases

GWAS have become one of the most important genetic methods for analyzing complicated trait QTLs and underlying genes. Many studies have shown that GWAS can be used to map more authentically new genes implicated in complex agronomic variables in plants. Given this, linkage disequilibrium (LD), population substructure, and imbalanced allele frequencies are the key drawbacks of GWAS. Many markers associated with tolerance to abiotic stresses have been also reported in chickpea. In brief, the germplasm of 186 chickpea genotypes has been genotyped with 1856 DArTseq markers. The association with the salinity tolerance in the field (Arish, Sinai, Egypt) and the greenhouse by using hydroponic system at 100 mM NaCl concentration indicated one locus on chromosome Ca4 at 10,618,070 bp associated with salinity tolerance, in addition to another locus-specific to the hydroponic system on chromosome Ca2 at 30,537,619 bp. The gene annotation analysis revealed the location of rs5825813 within the Embryogenesis-associated protein (EMB8-like), while the location of rs5825939 is within the Ribosomal Protein Large P0 (RPLP0) (Ahmed et al., 2021). Utilizing such markers in practical breeding programs can effectively improve the adaptability of current chickpea cultivars in saline soil.

Besides the above-mentioned reports, GWAS has also been conducted for yield and related traits in chickpea (Li et al., 2021), root morphological traits (Thudi et al., 2021), nutrient content (Diapari et al., 2014; Sab et al., 2020) and abiotic tolerance traits (Thudi et al., 2014; Samineni et al., 2022). Thus, the associated genomic regions identified through GWAS could be used for breeding programs to improve yield-related traits, nutrient content, and biotic and abiotic stress tolerance in chickpea. Recently, in other studies, we have accomplished GWAS for nodule numbers in chickpea by conducting multi-locational phenotypic evaluations and have identified seven significant SNP IDs (Kumar et al. unpublished data).

#### 3.2.11 Genetic Engineering for Broadening Genetic Bases

Genetic engineering has been widely utilized to select resistant gene(s) (Table 6) from various resources and transmit them to selected plants to introgress resistance to various abiotic as well as biotic challenges. Various genes are now being deployed in pulses using *Agrobacterium*-mediated (Eapen et al., 1987; Krishnamurthy et al., 2000; Sharma K. K. et al., 2006), particle gun bombardment (Kamble et al., 2003; Indurker et al., 2007), electroporation of intact axillary buds (Chowrira et al., 1996) electroporation and PEG mediated transformation using protoplasts (Köhler et al., 1987a; Köhler et al., 1987b). The most widely used method for developing transgenics in pulse crops is Agrobacterium mediated explant transformation. To generate transgenic plants, numerous transgenes from various sources have been introduced into pulse crops.

**TABLE 6:** List of engineered genes/traits in chickpea.

Crops	Genotype	Explant	Transgene	Promoter	Gene delivery system	Aim	References
Chickpea	C 235, BG 256, Pusa 362 and Pusa 372	Cotyledonary node	*cry1Ac*	*CaMV35S*	Agrobacterium-mediated	Insect resistance against *H. armigera*	Sanyal et al. (2005)
	ICCC37	Epicotyl	*cryIAc*	*CaMV35S*	Agrobacterium-mediated	Insect resistance against *H. armigera*	Indurker et al. (2007)
	Annigeri	Cotyledonary node	*P5CS*	*CaMV35S*	Agrobacterium-mediated	Salinity tolerance	Ghanti et al. (2011)
	P-362	Cotyledonary node	*cry1Ab* and *cry1Ac*	*CaMV35S* and synthetic constitutive expression promoter (*Pcec*)	Agrobacterium-mediated	Insect resistance	Mehrotra et al. (2011)
	DCP 92–3	Embryonic axis	*cry1Ab/cry1Ac*	Rice *actin1* and soybean *msg*	Agrobacterium-mediated	Insect resistance	Ganguly et al. (2014)
	Gokce	Mature embryo	*miR408*	CaMV35S	Agrobacterium-mediated	Drought tolerance	Hajyzadeh et al. (2015)
	ICCV 89,314	Single cotyledon with half embryo	*cry1Ac*	*RuBisCO* small subunit and ubiquitin	Agrobacterium-mediated	Insect resistance to target *H. armigera*	Chakraborty et al. (2016)
	DCP 92–3	Axillary meristem	*cry1Aabc*	*CaMV35S*	Agrobacterium-mediated	Insect resistance	Das et al. (2017)
	PBA HatTrick	Half-embryonic axis	nicotianamine synthase 2 and ferritin	*CaMV35S* and nopaline synthase	Agrobacterium-mediated	Iron biofortifcation	Tan et al. (2018)

Transgenic chickpea is developed either by gene gun (Kar et al., 1997; Husnain et al., 2000; Tewari-Singh et al., 2004; Indurker et al., 2007) or *Agrobacterium*-mediated method (Kar et al., 1997; Sanyal et al., 2005; Biradar et al., 2009; Acharjee et al., 2010; Asharani et al., 2011; Mehrotra et al., 2011; Ganguly et al., 2014). Important target traits for transgenic plant development in chickpea are insect pest resistance including α amylase inhibitor genes and lectin genes (Dita et al., 2006), Cry genes from *Bacillus thuringiensis*, protease inhibitor genes, disease resistance including transfer of genes such as chitinase gene, antifungal protein genes or stilbene synthase gene for fungal resistance, coat protein genes of viruses for viral resistance and bacterial resistance from T_4_ lysozyme gene (Eapen, 2008), various abiotic stresses like salinity, drought, mineral toxicities, cold, temperature, etc., seed proteins, plant architecture, and RNA interference technology could be used to increase carotenoids and flavanoids by engineering metabolic pathways to decrease the effect of endogenous genes (Eapen, 2008).

As presented in Table 7 transformation through Agrobacterium with the *cry1Ab/Ac* gene in chickpea has resulted in resistance to *Helicoverpa armigera* (Lawo et al., 2008; Ganguly et al., 2014). Bombardment of calli with DNA-coated tungsten particles resulted in somatic embryogenesis and the subsequent generation of transgenic chickpea (Husnain et al., 2000). Other researchers have also reported on the use of transgenic chickpea as a drought-tolerant and pest-resistant cultivar (Bhatnagar-Mathur et al., 2009; Khatodia et al., 2014; Kumar et al., 2014).

**TABLE 7:** Genetic transformation of chickpea.

Genotype	Explant	Transgene	Promoter	Gene delivery system	Aim	References
C 235, BG 256, Pusa 362 and Pusa 372	Cotyledonary node	*cry1Ac*	*CaMV35S*	Agrobacterium-mediated	Insect resistance against *H. armigera*	Sanyal et al. (2005)
ICCC37	Epicotyl	*cryIAc*	*CaMV35S*	Agrobacterium-mediated	Insect resistance against *H. armigera*	Indurker et al. (2010)
Annigeri	Cotyledonary node	*P5CS*	*CaMV35S*	Agrobacterium-mediated	Salinity tolerance	Ghanti et al. (2011)
P-362	Cotyledonary node	*cry1Ab* and *cry1Ac*	*CaMV35S* and synthetic constitutive expression promoter (*Pcec*)	Agrobacterium-mediated	Insect resistance	Mehrotra et al. (2011)
DCP 92–3	Embryonic axis	*cry1Ab/cry1Ac*	Rice *actin1* and soybean *msg*	Agrobacterium-mediated	Insect resistance	Ganguly et al. (2014)
Gokce	Mature embryo	*miR408*	CaMV35S	Agrobacterium-mediated	Drought tolerance	Hajyzadeh et al. (2015)
ICCV 89,314	Single cotyledon with half embryo	*cry1Ac*	*RuBisCO* small subunit and ubiquitin	Agrobacterium-mediated	Insect resistance to target *H. armigera*	Chakraborty et al. (2016)
DCP 92–3	Axillary meristem	*cry1Aabc*	*CaMV35S*	Agrobacterium-mediated	Insect resistance	Das et al. (2017)
PBA HatTrick	Half-embryonic axis	nicotianamine synthase 2 and ferritin	*CaMV35S* and nopaline synthase	Agrobacterium-mediated	Iron biofortification	Tan et al. (2018)

#### 3.2.12 Bioinformatic Molecular Data Bases/Resources for Broadening Genetic Bases

The recent data reports on leguminous genomics and transcriptomics have forced the creation of an exhaustive model of legume genomics and transcriptomics databases. Readily available data through online database portals are playing a significant role in research and development. LegumeIP (http://plantgrn.noble.org/LegumeIP/), an integrative database for comparative genomics and transcriptomics of model legumes, for use in studying gene function and genome evolution in this center-stage plant family including the genome sequences of *M. truncatula*, *G. max* and *L. japonicas* and two reference plant species, i.e., *A. thaliana* and *Populus trichocarpa* were employed (Li et al., 2012). The Legume Information System (LIS; https://legumeinfo.org) (Dash et al., 2016) gives users access to genetic and genomic data for model legumes. KnowPulse (https://knowpulse.usask.ca) for chickpea, common bean, field pea, fababean, and lentil, focuses on diversity data and gives information on germplasm, genetic markers, sequence variants, and phenotypic traits (Sanderson et al., 2019).

The construction of bioinformatics databases (Table 8) for the chickpea gene pool, according to recent breakthroughs in computational genomics, will permit users to visualize and extract chickpea genomics data in order to learn comparative genomics, annotate gene function, and investigate novel transcription factors (Doddamani et al., 2015; Verma et al., 2015; Gayali et al., 2016). Many databases have been built for chickpea, including CicArMiSatDB (https://cegresources.icrisat.org/CicArMiSatDB/) for SSR markers (Doddamani et al., 2014), CicArVarDB (https://cegresources.icrisat.org/cicarvardb/) for SNPs and QTLs, and Chickpea Transcriptome Database (Verma et al., 2015). Furthermore, a few years ago, the PLncPRO tool was developed to acquire unique insights into the rising importance of long noncoding RNAs in response to various abiotic challenges in chickpea (Singh et al., 2017).

**TABLE 8:** Bioinformatics resources for chickpea.

Bioinformatics resources for chickpea	Description
1. CicArMiSatDB (https://cegresources.icrisat.org/CicArMiSatDB/)	CicArMiSatDB is a web resource for learning about Chickpea microsatellite (Simple Sequence Repeat) markers. It gives the chickpea breeding community useful marker information. This database can be used to find marker information and examine it using the BLAST and Genome Browser implementations
2. PulseDB (https://www.pulsedb.org/organism/641)	The Pulse Crop Database (PCD), formerly the Cool Season Food Legume Database (CSFL), is being developed by Washington State University’s Main Bioinformatics Laboratory in collaboration with the USDA-ARS Grain Legume Genetics and Physiology Research Unit, the USDA-ARS Plant Germplasm Introduction and Testing Unit, the United States Dry Pea and Lentil Council, Northern Pulse Growers, and allied scientists in the United States and around the world, to serve as a resource for (GAB). By providing relevant genomic, genetic, and breeding information and analysis, GAB provides tools to find genes associated with features of interest, as well as other approaches to increase plant breeding efficiency and research
3. ACPFG Bioinformatics TAGdb (http://sequencetagdb.info/tagdb/cgi-bin/index)	This service performs BLAST alignment between a single query and short pair reads of selected species
4. The chickpea portal (http://www.cicer.info/)	In collaboration with partners in India (ICRISAT), this AISRF-funded project is focused on the development of efficient selection methods for tolerance to abiotic stress and the application of molecular tools to assist chickpea breeding
5. LIS ChickpeaMine (https://mines.legumeinfo.org/chickpeamine/begin.do)	This mine integrates data for chickpea varieties desi and kabuli. It is developed by LIS/NCGR and sourced from LIS datastore files
6. Chickpea Transcriptome Database (CTDB) (http://nipgr.res.in/ctdb.html)	It provides a full web interface for visualizing and retrieving chickpea transcriptome data. Many tools for similarity searches, functional annotation (putative function, PFAM domain, and gene ontology) searches, and comparative gene expression analyses are included in the database. The latest version of CTDB (v2.0) contains transcriptome datasets from farmed (desi and Kabuli kinds) and wild chickpea with high-quality functional annotation

There are also other molecular databases developed in other pulse crops which are useful in comparative genomics studies. Some of the important databases are highlighted as further. The PIgeonPEa Microsatellite DataBase (PIPEMicroDB) program (http://cabindb.iasri.res.in/pigeonpea/) stores a catalogue of microsatellites retrieved from the pigeon pea genome (Sarika et al., 2013). The adaptation of this program for chromosome-based search may be utilized for QTL markers for crop improvement and mapping of genes. With the fast development of publicly available Affymetrix GeneChip Medicago Genome Array Gene Chip data from cell types, a wide range of tissues, growth conditions, and stress treatments, the legume research group is in need of an efficient bioinformatics system to assist efforts to analyze the *Medicago* genome through functional genomics. The MtGEA (*Medicago truncatula* Gene Expression Atlas) website (http://bioinfo.noble.org/gene-atlas/) now includes additional gene expression data and genome annotation (He et al., 2009). The *Medicago truncatula* Genome Database (http://www.medicagogenome.org) houses a diverse collection of genomic data sets (Krishnakumar et al., 2015). RNA-Seq Atlas (Seq-Atlas) for *Glycine max* (http://www.soybase.org/soyseq) gathers RNASeq data from a variety of tissues and offers new techniques for analyzing huge transcriptome data sets produced from next-generation sequencing (Severin et al., 2010). SoyBase (https://www.soybase.org/), the USDA-ARS soybean genetic database, is a comprehensive library of professionally maintained soybean genetics, genomics, and related data resources (Grant et al., 2010). The *Lotus japonicus* Gene Expression Atlas (LjGEA: http://ljgea.noble.org/) provides a global picture of gene expression in organ systems of the species including roots, nodules, stems, petioles, leaves, flowers, pods, and seeds. It enables versatile, multifaceted transcriptome analysis (Verdier et al., 2013).

#### 3.2.13 Genome Editing for Broadening Genetic Bases

Genome editing promises giant leaps forward in broadening the genetic bases research. Targeted DNA integration into known locations in the genome has potential advantages over the random insertional events typically achieved using conventional means of genetic modification. The gene of interest is positioned near the T-DNA left border which is responsible for the insertion of plant cell. Molecular biologists can now more accurately target any gene of interest because advances in genome editing tools such as zinc-finger nucleases (ZFNs), homing endonuclease and transcription activator-like effector nucleases (TALENs) could possibly be exploited for genomics-assisted selection toward accelerated genetic gains (Shan et al., 2013; Bortesi and Fischer, 2015), while more advancements in chickpea enhancement using these cutting-edge approaches are still awaited. In chickpea, the 4-coumarate ligase (4CL) and Reveille 7 (RVE7) genes were selected as genes associated with drought tolerance for CRISPR/Cas9 editing in chickpea protoplast. The knockout of these selected genes in the chickpea protoplast showed high-efficiency editing was achieved for RVE7 gene *in vivo* compared to the 4CL gene (Badhan et al., 2021). These methods, however, are costly and time-consuming since they need complex procedures that require protein engineering. Unlike first-generation genome editing techniques, CRISPR/Cas9 genome editing is straightforward to design and clone and the same Cas9 can theoretically be used with various guide RNAs targeting many places in the genome. Several proof-of-concept demonstrations in crop plants using the primary CRISPR-Cas9 module, and numerous customized Cas9 cassettes have been used to improve target selectivity and reduce off-target cleavage. Thus, the applications of genome editing techniques in chickpea research have great potential (Mahto et al., 2022).

## 4 Integrating Various Omics Approaches for Broadening the Chckpea Genetic Base

**FIGURE 5:**
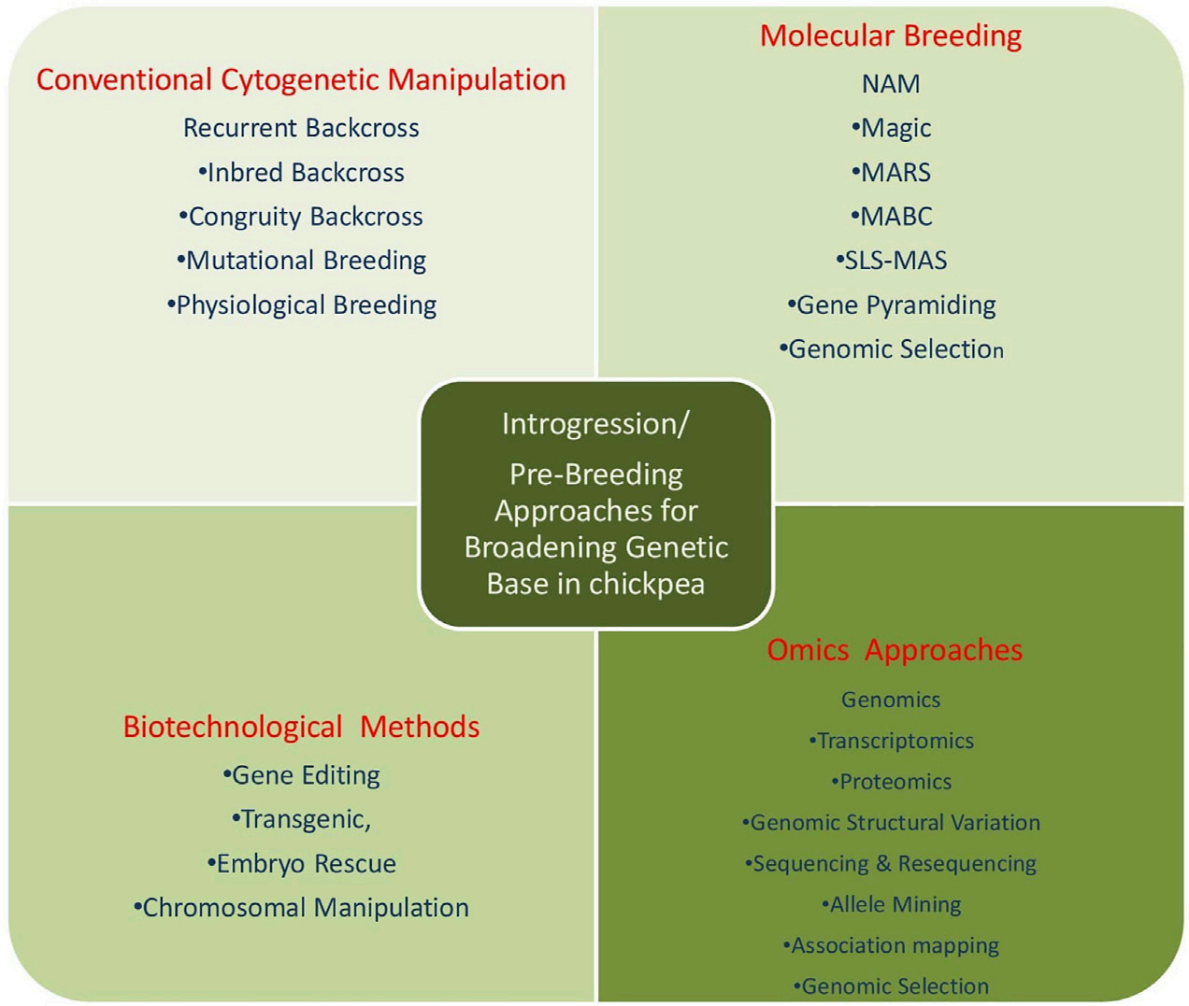
Integrating various approaches for broadening the genetic base.

The technological advances that transformed chickpea from an orphan crop to a genomic resource enriched crop in the post-genomics era, Re-sequencing efforts using WGRS have led to the dissection of genetic diversity, population structure, domestication patterns, linkage disequilibrium and the unexploited genetic potential for chickpea improvement (Varshney et al., 2019). Modern genomics technologies have the potential to speed up the process for trait mapping, gene discovery, marker development and molecular breeding, in addition to enhancing the rate of productivity gains in chickpea. Integration of genome-wide sequence information with precise phenotypic variation allows capturing accessions with low-frequency variants that may be responsible for essential phenotypes such as yield components, abiotic stress tolerance, or disease resistance (Roorkiwal et al., 2020). NGS technology has resulted in the development and application of a wide variety of molecular markers for chickpea improvement (Kale et al., 2015; Varshney et al., 2018). Over the past decade, more than 2000 simple sequence repeat (SSR) markers, 15,000 features-based diversity array technology (DArT) platform, and millions of SNP markers have been developed for chickpea (Varshney 2016). The revolution in NGS technologies has enabled sequencing to be performed at a higher depth (whole-genome re-sequencing), mid-depth (skim sequencing), or lower depth (genotyping by sequencing, RAD-Seq). Integrating omics data from multiple platforms such as transcriptomics, proteomics and metabolomics are paramount to bridging the genome-to-phenome gap in crop plants and ultimately identifying the phenotype based on their genetics. applications of genomic technologies for bridging the genotype–phenotype gap in chickpea (Figure 5). With the availability of the reference genome, these genetic resources can be subjected to whole-genome re-sequencing (WGRS) or high- to low-density genotyping, based on the objective of the study, using the available genotyping platforms (e.g., genotyping by sequencing, GBS; array-based genotyping). Analysis at the transcriptome, proteome, and metabolome levels can be performed to gain novel insights into the candidate genes and biological processes involved. Using a genomics approach Fusarium wilt resistance WR 315 Annigeri 1 foc4 has been Released as “Super Annigeri 1′ for commercial cultivation in India Mannur et al. (2019).

## 5 Conclusion and Future Perspective

With the employment of modern “Omics” technologies in combination with traditional methods, it is now possible to overcome yield limits, and achieve higher genetic gains ensuring high output for chickpea production and quality features. Chickpea land races and wild *Cicer* species are the goldmines of beneficial genes influencing desired traits of interest for biotic, abiotic, and yield component features. Identification of novel sources of desired traits, QTLs or alleles through extensive evaluation and utilization of landraces and wild *Cicer* species will have a greater impact on developing chickpeas for better climate resilience and higher yield. Many desirable features from primary and secondary gene pools in wild *Cicer* species have been successfully transmitted into cultivated cultivars using both traditional and modern procedures. The wealth of new omics approaches and growing resources offer great potential to transform chickpea breeding in the near future. An integrated application of chickpea “Omics”, classical and modern breeding methods, marker-assisted selection, and biotechnological application promises for the broadening of the chickpea genetic base and introgression of new genes for crop traits for higher productivity will lead to next-generation chickpea varieties.

## Data Availability

The original contributions presented in the study are included in the article/Supplementary Material, further inquiries can be directed to the corresponding author.
